# Transfer of Proteins from Cultured Human Adipose to Blood Cells and Induction of Anabolic Phenotype Are Controlled by Serum, Insulin and Sulfonylurea Drugs

**DOI:** 10.3390/ijms24054825

**Published:** 2023-03-02

**Authors:** Günter A. Müller, Timo D. Müller

**Affiliations:** 1 Institute for Diabetes and Obesity (IDO), Helmholtz Diabetes Center (HDC) at Helmholtz Zentrum München, German Research Center for Environmental Health (GmbH), Ingolstädter Landstraße 1, 85764 Oberschleissheim, Germany; 2 German Center for Diabetes Research (DZD), 85764 Oberschleissheim, Germany

**Keywords:** diabetes, glimepiride, glycosylphosphatidylinositol (GPI)-anchored proteins (GPI-APs), (G)PI-specific phospholipase D (GPLD1), insulin action, protein transfer, sulfonylurea drugs (SUs)

## Abstract

Glycosylphosphatidylinositol-anchored proteins (GPI-APs) are anchored at the outer leaflet of eukaryotic plasma membranes (PMs) only by carboxy-terminal covalently coupled GPI. GPI-APs are known to be released from the surface of donor cells in response to insulin and antidiabetic sulfonylureas (SUs) by lipolytic cleavage of the GPI or upon metabolic derangement as full-length GPI-APs with the complete GPI attached. Full-length GPI-APs become removed from extracellular compartments by binding to serum proteins, such as GPI-specific phospholipase D (GPLD1), or insertion into the PMs of acceptor cells. Here, the interplay between the lipolytic release and intercellular transfer of GPI-APs and its potential functional impact was studied using transwell co-culture with human adipocytes as insulin-/SU-responsive donor cells and GPI-deficient erythroleukemia as acceptor cells (ELCs). Measurement of the transfer as the expression of full-length GPI-APs at the ELC PMs by their microfluidic chip-based sensing with GPI-binding α-toxin and GPI-APs antibodies and of the ELC anabolic state as glycogen synthesis upon incubation with insulin, SUs and serum yielded the following results: (i) Loss of GPI-APs from the PM upon termination of their transfer and decline of glycogen synthesis in ELCs, as well as prolongation of the PM expression of transferred GPI-APs upon inhibition of their endocytosis and upregulated glycogen synthesis follow similar time courses. (ii) Insulin and SUs inhibit both GPI-AP transfer and glycogen synthesis upregulation in a concentration-dependent fashion, with the efficacies of the SUs increasing with their blood glucose-lowering activity. (iii) Serum from rats eliminates insulin- and SU-inhibition of both GPI-APs’ transfer and glycogen synthesis in a volume-dependent fashion, with the potency increasing with their metabolic derangement. (iv) In rat serum, full-length GPI-APs bind to proteins, among them (inhibited) GPLD1, with the efficacy increasing with the metabolic derangement. (v) GPI-APs are displaced from serum proteins by synthetic phosphoinositolglycans and then transferred to ELCs with accompanying stimulation of glycogen synthesis, each with efficacies increasing with their structural similarity to the GPI glycan core. Thus, both insulin and SUs either block or foster transfer when serum proteins are depleted of or loaded with full-length GPI-APs, respectively, i.e., in the normal or metabolically deranged state. The transfer of the anabolic state from somatic to blood cells over long distance and its “indirect” complex control by insulin, SUs and serum proteins support the (patho)physiological relevance of the intercellular transfer of GPI-APs.

## 1. Introduction

Soon after the discovery that certain cell surface proteins are anchored at the outer leaflet of the plasma membranes (PMs) of eukaryotic cells by a glycosylphosphatidylinositol (GPI) glycolipid that is covalently bound to the carboxy terminus of the protein moiety [1,2], the question concerning the role of membrane anchorage by GPI arose. Approximately 200 GPI-anchored proteins (GPI-APs) have been predicted or identified so far in mammalian cells, based on the carboxy-terminal consensus sequence for GPI addition and biochemical analysis, respectively [3,4]. The highly conserved GPI consists of amphiphilic phosphatidylinositol with two long-chain (mostly saturated) fatty acyl chains and a hydrophilic glycan core composed of glycosidically linked nonacetylated glucosamine and three mannose residues, which are amide-linked to the carboxy-terminus of the protein moiety via an ethanolamine-phosphodiester bridge (for structural details see Supplementary Materials, Supplementary Figure S1). GPI-APs are assembled along the secretory pathway (for a review, see [5,6,7]).

Low space requirement at PMs, increased lateral mobility within the plane of the outer PM leaflet, capability for accumulation at higher density, in particular at certain cell surface microdomains, such as lipid rafts [8] (for a review, see [9,10,11,12]), and the possibility of the specific, rapid and controlled release from the cell surface by a single type of enzyme, GPI-specific phospholipases (GPI-PLs; for a review, see [13,14,15,16]), rather than by a multitude of different proteases, have been regarded as potential main reasons for the cell surface anchorage of proteins via GPI rather than by typical proteinaceous transmembrane domains. The functional implications of GPI anchorage apparently differ for each GPI-AP, since the exchange of a GPI for a transmembrane anchor by recombinant technology has been demonstrated to result in complete loss, impairment only or even maintenance of the function for the GPI-APs studied [17,18,19,20].

Considering the controlled release of GPI-APs, a multitude of GPI-APs, among them lipoprotein lipase, glycolipid-anchored cAMP-binding/phosphodiesterase ectoprotein Gce1 and 5’-nucleotidase CD73 [21,22,23], have been demonstrated to be lipolytically cleaved from the surface of a variety of eukaryotic cells, such as rat primary and cultured (3T3-L1) adipocytes, under certain (patho)physiological conditions or in response to certain hormones and stimuli, among them glucose [21,24], insulin [23,25,26,27] and antidiabetic sulfonylureas (SUs) [22,23,28]. In particular, the treatment of primary or cultured rodent adipocytes, cultured RAW264 or microglial cells with pharmacological concentrations of the antidiabetic SU of the 3rd generation, glimepiride (for structural details see Supplementary Materials, Supplementary Figure S2), a potent amphiphilic-to-hydrophilic conversion and release from the cell surface as soluble proteins were observed for a subset of GPI-APs, such as Gce1 and CD14, respectively, which is due to the activation of an endogenous GPI-PLC, with its catalytic ectodomain exposed at the cell surface [21,22,23,24,25,26,27,28]. The identity of the mammalian GPI-PLC(s), in particular the insulin- and SU-dependent one(s), remains to be elucidated as holds true for the molecular mechanism(s) of their activation, albeit tyrosine kinase signaling [29] and glucose transport-dependent [21,24] pathways have been suggested.

Experimental findings obtained over the last two decades have increasingly focused on the apparently labile cell surface anchorage of GPI-APs with resulting amphitropic localization at both cell surfaces and in extracellular compartments, as a consequence of their spontaneous nonenzymic release from the outer leaflet of PMs due to the fact of their pronounced overall amphiphilic character (i.e., large hydrophilic protein moiety coupled to amphiphilic GPI) (for a review, see [15,16]). The capability of full-length GPI-APs for spontaneous release from and insertion into PM, as deduced from this large body of experimental data obtained in vitro, immediately raised the possibility of a physiological function of the intercellular transfer of GPI-APs in multicellular (e.g., mammalian) organisms between donor and acceptor cells of the same type over a short distance (i.e., within the same tissue depot) and in the so-called “direct” mode, i.e., without interference by other (e.g., serum) proteins. In fact, “direct” transfer has recently been demonstrated for large heavily lipid-loaded and small less lipid-loaded adipocytes [30], which may shift the burden of lipid synthesis from the former to the latter in case of the need of excessive fat deposition in adipose tissues. In contrast, GPI-AP transfer between different cell types over a long distance (i.e., between different tissue depots) has been considered as unwanted: transfer of GPI-APs typically covering a broad spectrum of functions, such as receptors, binding proteins, enzymes, cell adhesion, and matrix proteins [5,7,13], across extracellular compartments, such as interstitial spaces and blood, to acceptor cells of different tissue depots, which do not express them at the surface in the normal state, could lead to deleterious effects. Consequently, it has been postulated that mammalian organisms had to develop strategies to prevent the transfer of GPI-APs between cells over a long distance via the circulation. In agreement, upregulated cleavage by the major serum protein, GPI-specific phospholipase D (GPLD1) [31,32], and interaction with serum proteins of the GPI of GPI-APs have been reported [33,34] and correlated to the elevated release of the latter in response to adverse effectors, such as age and metabolic state, thereby blocking their transfer in the so-called “indirect” mode, i.e., with the aid of other (e.g., serum) proteins.

In the present study, a putative role of the controlled lipolytic release of GPI-APs from donor cells with loss of their GPI anchor in concert with serum GPI-binding proteins in the prevention of GPI-AP transfer to and associated effects in acceptor cells different and distant from the donor cells was investigated.

For this, the previously introduced transwell co-culture of differentiated human adipocytes [30] as donor cells and mutant erythroleukemia K562 (EL) cells (ELCs), which fail to express GPI-APs at their surface [35], as acceptor cells and detection of the transferred GPI-APs with a chip-based microfluidic sensor system [36,37] were used. The latter relies on the propagation of surface acoustic waves (SAWs) along the chip surface, which is affected in the course of binding to the chip of any entities, in general, and interaction between partners of interest, in particular, one of which, here PMs, became immobilized onto the chip surface with the aid of ionic and covalent capturing procedures. The other one, binding-proteins against the immobilized partner, here α-toxin for the GPI anchor (for a review, see [38]) or antibodies against the protein moieties of the transferred GPI-APs, is subsequently injected into the chip channels (single or in sandwich). The resulting rightward phase shift (i.e., increase in difference in phase or decrease in frequency) of the SAWs represents a real-time measure for the mass loaded, here the nature and amount of full-length GPI-APs that were transferred during the previous incubation of the ELCs, onto the immobilized sample analyte, here their PMs.

Both insulin and glimepiride caused the downregulation of the transfer of GPI-APs from human adipocytes to ELCs and the accompanying stimulation of glycogen synthesis. Unexpectedly, in the presence of serum insulin and glimepiride this led to increased GPI-AP transfer and glycogen synthesis, dependent on the metabolic state of the rats, from which the serum was derived. The differential serum-dependent effects of insulin and glimepiride on GPI-AP transfer were shown to rely on the displacement of full-length GPI-APs from serum GPI-binding proteins by GPI-APs lipolytically cleaved in response to insulin or SUs.

Taken together, the present study suggests that the transfer of GPI-APs between different and distant cells (e.g., from donor adipose to acceptor blood cells) may exert (patho)physiological roles in the acceptor cells (e.g., stimulation of glycogen synthesis), which is under the “indirect” control of a complex interplay of serum proteins (e.g., GPLD1), signals (e.g., insulin) and drugs (e.g., SUs).

## 2. Results

### 2.1. Stimulation of Basal Glycogen Synthesis upon Transfer of GPI-APs Depends on Their Localization at the PMs of the Acceptor ELCs

**Figure 1:**
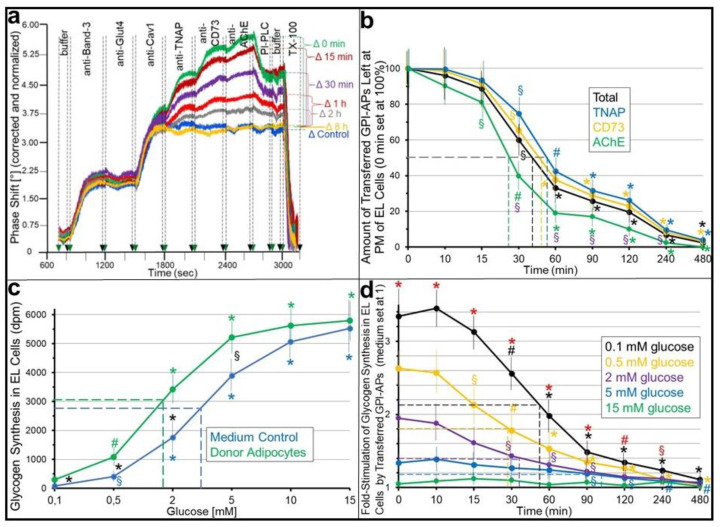
Parallel loss of transferred GPI-APs and of upregulated glycogen synthesis in acceptor ELCs. Transwell co-cultures were run with human adipocytes (of lipid-loading stage IV) as donor cells or only medium (Control) and GPI-deficient ELCs as acceptor cells in the insert and bottom wells, respectively, as described in Section 4. (**a**) After incubation (i) for transfer (1 week, 37 °C, absence of serum and BSA), the insert wells were removed. The bottom wells were rinsed once with medium and then subjected to incubation (ii) with medium for internalization (37 °C, absence of serum and BSA) for the periods indicated (Control for 8 h). Subsequently, PMs were prepared from the ELCs of the bottom wells, then coupled to chips by ionic/covalent capture and, finally, analyzed for the expression of membrane proteins by SAW sensing, as described in Section 4. Phase shifts induced by the sequential binding of antibodies against transmembrane proteins (800–1800 s) and then GPI-APs (1800–2700 s) and by the subsequent action of bacterial PI-PLC (2700–2900 s) and, finally, TX-100 (3000–3200 s) are shown, omitting the preceding capturing of the PMs (0–700 s; for details as well as methods of correction and normalization, see [29]). Phase shift Δ measured in response to the injection of the anti-TNAP, CD73 and AChE antibodies (1800–2700 s) as summation signals, indicated by the horizontal, hatched lines and brackets for each incubation period. (**b**) The experiment was repeated four to six times (distinct incubations for both [i] and [ii]). Relative amounts of total GPI-APs (black line, summation signals corresponding to panel a, 1800–2700 s) or individual GPI-APs (colored lines, single signals corresponding to panel a, 1800–2100, 2100–2400, 2400–2700 s) transferred from the adipocytes during the incubation (i) and left at PMs of ELCs after the various periods of incubation (ii) are given. Significant differences between start of internalization (100% GPI-APs left at PMs at 0 min of incubation [i]) and the various periods of second incubation (0–480 min) for both total (black symbols) and individual GPI-APs (colored symbols), as well as between total GPI-APs and AChE, for each period of incubation (ii) (turquois symbols) are indicated. (**c**,**d**) The acceptor ELCs, which were run for 120 min (**c**) or increasing periods (**d**) of incubation, (ii) were used for the determination of glycogen synthesis during incubation (iii) (15 min, 37 °C, presence of BSA) with [U-^14^C]glucose at increasing concentrations and a constant specific radioactivity, as described in Section 4. (**c**) The absolute amount of glycogen synthesized in the ELCs is given (dpm) for the configuration with donor adipocytes or only medium as the control in the insert wells, with significant differences between glucose at 0.1 mM and higher concentrations (green and blue symbols, respectively), as well as between donor adipocytes and medium control, at each glucose concentration (black symbols) indicated. (**d**) The fold-stimulation of glycogen synthesis in ELCs is given for the configuration with donor adipocytes in the insert wells (medium control set at 1) and for each glucose concentration and each period of incubation (ii). Significant differences between 0 min and the various periods of internalization are indicated for each glucose concentration (correspondingly colored symbols), as well as between 0.1 and 15 mM glucose for each period of internalization (red symbols) (means ± SD; * *p* ≤ 0.01, # *p* ≤ 0.02, ^§^ *p* ≤ 0.05). (**b**,**d**) Half-life times for 50% loss of transferred GPI-APs from the PMs of ELCs and 50% reduction of glycogen synthesis stimulation (as indicated by the horizontal, hatched lines) are given by the vertical, hatched lines. (**c**) Glucose concentrations for the half-maximal stimulation of glycogen synthesis in ELCs (as indicated by the horizontal, hatched lines) are given by the vertical, hatched lines.

Full-length GPI-APs with the complete GPI anchor remaining attached become released from and inserted into the outer leaflets of PMs of human donor adipocytes and acceptor ELCs, respectively, with the accompanying upregulation of glycogen synthesis in the latter, as recently demonstrated using transwell co-culture and SAW sensing [29]. However, it has been left open whether the transferred GPI-APs remain localized at the PMs in order to exert their putative biological function. The extra- vs. intracellular localization of the transferred GPI-APs, in particular their possible internalization in the acceptor cells, which has been amply documented with native GPI-APs after their biogenesis in various cell types, was assayed with GPI-deficient ELCs as acceptor cells, which facilitate the detection of the putative physiological effects of GPI-AP transfer due to the missing background of endogenous GPI-APs (for a more detailed explanation, see [30]). After incubation with human adipocytes as donor cells for the induction of the transfer (i) and further incubation in the absence of the donor adipocytes for increasing periods to provoke their internalization (ii), PMs were analyzed for the expression of the total and individual GPI-APs (iii; Figure 1a,b) and, in parallel, glycogen synthesis was measured at low (basal) and high concentrations of glucose (iv; Figure 1c,d).

For the determination of the rather low amounts of GPI-APs transferred during the transwell co-culture (for more details, see [30]), a chip-based microfluidic sensor system of very high sensitivity, accurateness and reproducibility, as well as robustness, towards the turbidity and viscosity caused by the lipidic entities in the samples was used. It relies on the propagation of SAWs along the gold surface of the chip channels, which is affected by any interaction of macromolecules with the channels, e.g., by capture of PMs through combined ionic and covalent bonds upon their injection, as well as subsequent binding of antibodies directed towards transmembrane proteins or GPI-APs expressed at those PMs. The resulting rightward phase shift (decrease in frequency) of the SAWs represents a measure for the mass (i.e., relevant antibodies) loaded onto the chip (as single entities or in sandwich) and, consequently, for the amount of a specific membrane protein at the PMs. Thus, both successful capture of the PMs, prepared from the acceptor cells, by the TiO_2_ chip surface (not shown here; for details, see [30]) and the nature and amount of specific GPI-APs expressed at the acceptor PMs after their (i) transfer to and (ii) eventual internalization by the acceptor ELCs during transwell co-culture together with donor adipocytes (i) and then alone (ii) can be monitored as increases in the phase shift.

The SAW sensing of the PMs of acceptor cells that were incubated with medium alone did not result in phase shift increases upon the injection of anti-TNAP, CD73 and AChE antibodies (Figure 1a, Δ Control, 1800–2700 s) in agreement with the lack of expression of those GPI-APs by GPI-deficient ELCs, as has been recently reported [35,39]. However, the phase shift Δ between incubation (i) (for one week) of the acceptor ELCs with (Figure 1a, Δ 0 min of incubation [ii]) but not without (Figure 1a, Δ Control) donor adipocytes increased in stepwise fashion, indicating the transfer of TNAP, CD73 and AChE from the PMs of donor adipocytes to the PMs of acceptor ELCs. By contrast, no phase shift Δ were measured for the transmembrane proteins Band-3 and Glut4, as well as the atypical caveolae-specific membrane protein Cav1, which were all endogenously expressed by the GPI-deficient ELCs but failed to be transferred from the human adipocytes during transwell co-culture incubation (i) (Figure 1a, Δ Control, 800–1800 s).

The expression of proteins at PMs is typically determined by the rates of their synthesis/exocytosis and internalization/degradation, which are at equilibrium during continuous cell growth. This holds true with the transmembrane proteins, since the antibody-induced phase shifts did not increase upon incubation (ii) of the ELCs with medium alone (i.e., upon the removal of the insert wells with the donor adipocytes from the bottom wells with the ELCs) for increasing periods (Figure 1a, Δ 0 min-8 h, 800–1800 s). By contrast, antibody-induced phase shift increases for the transferred GPI-APs declined over time of the incubation (ii) in the absence of the donor cells (Figure 1a, Δ 0 min-8 h, 1800–2700 s). Eight hours after the termination of the GPI-AP transfer, the phase shift reached control levels, as observed for the missing transfer upon incubation with medium alone (Figure 1a, Δ Control, 1800–2700 s). These data reflect the continuous internalization (during incubation [ii]) of the transferred GPI-APs in the GPI-deficient ELCs, which only became apparent upon the termination of the transfer (during incubation [i]), leading to a rightward shift of the steady state of the PM expression of GPI-APs between their transfer and internalization.

Bacterial PI-PLC specifically cleaves off the diacylglycerol moiety of GPI anchors from GPI-AP protein moieties. Moreover, it may release those PM vesicles from the chips, which have been immobilized through ionic/covalent capture of GPI-APs. In fact, the injection of bacterial PI-PLC (Figure 1a, 2700–2900 s) following that of the antibodies led to a decline in antibody-induced phase shifts in sandwich compatible with anchorage of the corresponding transferred and internalized proteins through full-length GPI. The only 30 to 50% reduction may be explained by partial lipolytic digestion only due to the fact of the impaired accessibility of the GPI-APs for bacterial PI-PLC, which is known to depend on the type of both cell and protein. Importantly, acylation at the 2-position of the *myo*-inositol residue of the glycan core of human GPI-APs, which occurs during early biogenesis (and becomes reversed during later stages for some but not all human cell types and GPI-APs, such as for erythrocyte AChE, decay accelerating factor and placental alkaline phosphatase), was reported to impair cleavage by bacterial PI-PLC [5,6,7,40,41]. An only partial regain of deacylase expression during prolonged culture of ELCs could be responsible for the removal of only a portion of the transferred GPI-APs, the deacylated ones, from PMs (Figure 1a, 2700–2900 s).

The portions of the phase shift increase corresponding to acylated GPI-APs, which were transferred (incubation [i]) yet not internalized (during the initial period of incubation [ii]; Δ 0 min–2 h) and resisted PI-PLC, were completely eliminated following the injection of TX-100 (Figure 1a, 3000–3200 s). This agreed with the solubilization of the captured PM and confirmed that any alteration in phase shift measured by SAW sensing relied on the PMs captured by the chips.

The quantitative evaluation of the number of GPI-APs left at the PMs of GPI-deficient ELCs immediately after the termination of transfer (after one week of incubation [i]; see Figure 1a, green curve, Δ 0 min) and after subsequent incubation (ii) for increasing periods (see Figure 1a, Δ 15 min–8 h) revealed the time-dependent loss from PMs, i.e., internalization of the total and individual transferred GPI-APs (Figure 1b). The kinetics revealed its completion after 6 to 8 h for all GPI-APs but with significant differences in the half-life times, ranging from 20 to 25 min for AChE to up to 50 to 55 min for TNAP.

Previous data have shown that the transfer of total adipocyte GPI-APs to GPI-deficient ELCs is correlated to the upregulation of basal glycogen synthesis, as measured at a low glucose concentration [30]. Importantly, this apparent biological effect of GPI-AP transfer is glucose-dependent and declined with increasing concentrations of glucose, as shown here by the transfer of GPI-APs from donor adipocytes (Figure 1c, 0 min, green lines) or control incubation (Figure 1c, medium, blue lines), subsequent incubation of the acceptor ELCs in the insert wells of the transwell co-culture with increasing concentrations of radiolabeled glucose (at identical specific radioactivity) and final assay for the amount of glycogen synthesized. As expected, radiolabeled glycogen increased with increasing concentrations of glucose in ELCs lacking, as well as harboring, transferred GPI-APs due to the fact of mass action. However, the stimulation of glycogen synthesis by the transferred GPI-APs was more pronounced at low (0.1–5 mM) glucose concentrations. This led to a leftward shift of the concentration–response curve for ELCs with transferred GPI-APs, as revealed by the lower glucose concentration effective for half-maximal stimulation (1.6 mM) compared to GPI-deficient ELCs (3.9 mM) (Figure 1c).

Next, the effect of the internalization of the transferred GPI-APs in ELCs on glucose-dependent glycogen synthesis was investigated (Figure 1d). The stimulation of glycogen synthesis by the transferred GPI-APs decreased with the increasing periods of incubation (ii) after the termination of transfer (incubation [i]) and start of the decline of the GPI-APs at PMs (Figure 1d, 0 min), irrespective of the glucose concentration present during the assay. The half-life times, as well as the time points, for the complete abrogation of glycogen synthesis stimulation (Figure 1d, 4–8 h) were like those for the internalization of the transferred GPI-APs (Figure 1b). Moreover, at the start of the internalization (zero time point), the fold-stimulation of glycogen synthesis (at identical, specific radioactivity) by transferred adipocyte GPI-APs was highest at 0.1 mM glucose and significantly decreased with increasing glucose concentrations to up to the complete loss of stimulation at 15 mM. In conclusion, the comparable kinetics of the internalization of the transferred GPI-APs and downregulation of (predominantly basal) glycogen synthesis in the acceptor ELCs argued for a mechanistic link between the transfer to and residence at their PMs of GPI-APs and sensitization of glycogen synthesis towards glucose.

**Figure 2:**
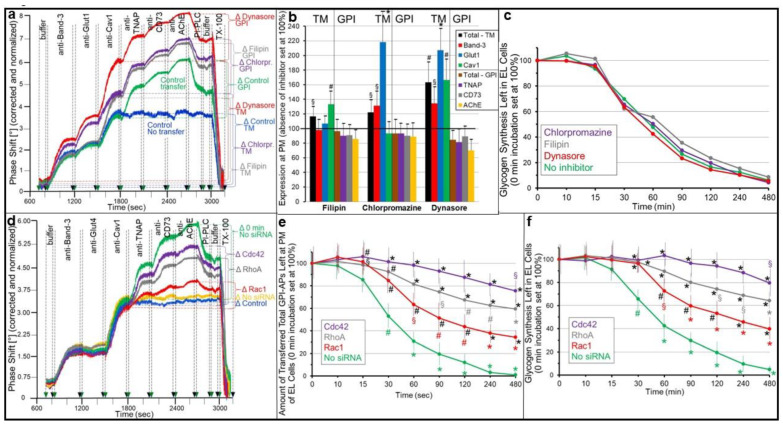
Inhibition of the internalization of transferred GPI-APs via the GEEC and persistence of transfer-induced glycogen synthesis in ELCs. Transwell co-cultures were run with human adipocytes (of lipid-loading stage IV) as donor cells or only medium (Control no transfer) and GPI-deficient ELCs as acceptor cells in the insert and bottom wells, respectively, as described in Section 4. (**a**) After incubation (i) for transfer (1 week, 37 °C, absence of serum and BSA), the insert wells were removed. The bottom wells were rinsed once with medium and then subjected to incubation (ii) with medium for internalization (480 min, 37 °C, absence of serum and BSA) in the absence (Control transfer) or presence of Dynasore (80 µg/mL), filipin (0.5 µg/mL) and chlorpromazine (50 µM). The following procedures were performed as described for Figure 1a. Phase shift Δ was measured in response to the injection of anti-Band-3, Glut1 and Cav1 (800–1800 s; TM), as well as TNAP, CD73 and AChE antibodies (1800–2700 s; GPI), as summation signals indicated by the horizontal, hatched lines and brackets for each incubation condition. (**b**) The experiment was repeated four and five times (distinct incubations [ii]). Relative amounts of the total (summation signals, black bars) and individual (colored bars) transmembrane proteins (TM) and GPI-APs (GPI) transferred from the human adipocytes during incubation (i) and left at the PMs of the ELCs after incubation (ii) in the presence or absence (set at 100% each) of various inhibitors of endocytosis are given. Significant differences between the absence and presence of each inhibitor for both the total (black bars) and individual (colored symbols) TM and GPI-APs are indicated. (**c**) The acceptor ELCs in the bottom wells of the transwell co-cultures were used for the determination of glycogen synthesis during incubation (iii) for glycogen synthesis (15 min, 37 °C, presence of BSA) with [U-^14^C]glucose (0.1 mM) after the various periods of incubation (ii) in the absence (green line) or presence of the various inhibitors, as described in Section 4. Relative glycogen synthesis left in ELCs is given, with no incubation (ii) (0 min) set at 100%. (**d**) Transwell co-cultures were run with human adipocytes as donor cells or only medium (Control) and ELCs as acceptor cells in the insert and bottom wells, respectively, as described in Section 4. After incubation (i) for transfer (1 week, 37 °C, absence of serum and BSA), the insert wells were removed. The bottom wells were rinsed once with medium and then subjected to incubation (ii) with medium for internalization (37 °C, absence of serum and BSA) in the absence of siRNA for 0 min (No siRNA; green curve) and 480 min (No siRNA; orange curve) or presence of siRNAs (30 nM) directed against Cdc42, RhoA and Rac1 for 480 min. The following procedures were performed as described for Figure 2a, except for injecting anti-Glut4 rather than Glut1 antibodies. (**e**) The experiment was repeated four to seven times (distinct incubations [ii]). Relative amounts of the total GPI-APs (summation signals) transferred from the human adipocytes during incubation (i) and then left at the PMs of ELCs after incubation (ii) in the presence or absence of siRNAs are given, with the 0 min incubations set at 100% each. Significant differences between each incubation period and the 0 min incubation (colored symbols), as well as between the absence and presence of each siRNA for each incubation period, are indicated (black symbols). (**f**) The acceptor ELCs in the bottom wells of the transwell co-cultures were used for the determination of glycogen synthesis during incubation (iii) (15 min, 37 °C, presence of BSA) with [U-^14^C]glucose (0.1 mM) after the various periods of incubation (ii) in the absence (green line) or presence of each siRNA. The relative glycogen synthesis in the ELCs is given with no incubation (ii) (0 min) set at 100%. Significant differences between each incubation period and the 0 min incubation (colored symbols), as well as between the absence and presence of each siRNA for each incubation period, are indicated (black symbols) (means ± SD; * *p* ≤ 0.01, # *p* ≤ 0.02, ^§^ *p* ≤ 0.05).

These findings prompted the investigation of the effects of the inhibition of the internalization of the transferred GPI-APs on their residence at the PMs in parallel to the basal glycogen synthesis in the acceptor ELCs. For this, small-molecule inhibitors of the endocytosis of typical or atypical transmembrane proteins involving either clathrin-coated vesicles (chlorpromazine, Dynasore) or caveolae (filipin, Dynasore) (Figure 2a–c) [42], as well as siRNAs directed towards components of the GPI-AP-enriched endosomal compartment (GEEC [42,43,44,45,46]) (Rac1, RhoA, Cdc42 [47]) engaged in endocytosis of GPI-APs (Figure 2d–f) were used. The downregulation by the siRNAs, alone or in combination, was demonstrated for the corresponding RNAs by qPCR (Müller and Müller, unpublished data) and proteins by SAW sensing (Supplementary Materials, Supplementary Figure S3, Supplementary Table S1) and found to be specific and efficient (by 82 to 89% and 65 to 78%, respectively). After the termination of the GPI-AP transfer (incubation [i]) by removal of the donor adipocytes from the acceptor ELCs in the transwell co-culture, the inhibitors were added to incubation (ii) (480 min) for putative interference with the internalization of the GPI-APs. As expected, an analysis of the PMs of the ELCs by SAW sensing for typical (Band-3, Glut1) or atypical (Cav1) transmembrane proteins (TMPs) revealed significant increases in phase shift (Figure 2a; 800–1800 s; summation signals) and, thus, expression at PMs (Figure 2b; calculated total and individual TMPs, presence vs. absence of inhibitor) of Band-3 and Glut1 (to up to 2.2-fold) in response to chlorpromazine and Dynasore, and of Cav1 (to up to 1.6-fold) in response to filipin and Dynasore. In contrast, filipin, chlorpromazine and Dynasore did not significantly affect the PM expression of the GPI-APs TNAP, CD73 and AChE (Figure 2a; 1800–2700 s, Figure 2b).

The measurement of the glycogen synthesis of the ELCs after increasing periods of internalization confirmed the time-dependent decline of the transfer-induced basal glycogen synthesis (see Figure 1b), with complete loss after 480 min (Figure 2c, green line), but it did not reveal any effect of filipin, chlorpromazine or Dynasore on the kinetics of glycogen synthesis downregulation (Figure 2c).

The presence of siRNAs directed towards Rac1, RhoA and Cdc42 compared to their absence (Figure 2d; orange curve) after the termination of transfer during internalization led to significant upregulation of the amounts of transferred GPI-APs left at PMs, in this ranking order of increasing efficacy of the siRNAs (Figure 2d). The time courses of internalization, which were calculated as the number of transferred total GPI-APs left at PMs of the ELCs, were shifted to the right by the siRNAs, with those directed towards Cdc42 and Rac1 being most and least efficient, respectively (Figure 2e). After 480 min of incubation with Rac1, RhoA and Cdc42, approximately 40, 60 and 80% of the transferred total GPI-APs, respectively, was left at PMs compared to the absence of siRNAs (Figure 2d,e; green lines).

Importantly, the time courses of the internalization of the GPI-APs (Figure 2e) and downregulation of the glycogen synthesis (Figure 2f) in the ELCs in the course of silencing were similar, with rightward shifts by 15 to 30 min for both and Cdc42 being most effective in interfering with both. Importantly, silencing of Rac1, RhoA and Cdc42 did not affect expression at PMs of Band-3, Glut1 and Cav1 (Figure 2d; 800–1800 s) compared to “no-transfer” (Figure 2d; blue curve) and no-siRNA (Figure 2d; orange curve) control. Together the data (Figure 1 and Figure 2) imply that the physiological function of transferred GPI-APs does not depend on their endocytosis.

### 2.2. Insulin and Antidiabetic SUs Inhibit Both GPI-AP Transfer to and Transfer-Induced Glycogen Synthesis in Acceptor ELCs

Previous research [30,48] and the above data have demonstrated that the intercellular transfer of GPI-APs and the accompanying stimulation of anabolic processes (glycogen and lipid synthesis) in the acceptor cells critically depend on the preservation of the complete GPI anchor of the GPI-APs, as well as their localization at the PMs of the acceptor cells. Insulin and the antidiabetic SUs of the 2nd and 3rd generation, glibenclamide and glimepiride (for the structure of typical representatives, see Supplementary Materials, Supplementary Figure S2), are known to release GPI-APs from the surface of insulin target cells, such as primary and cultured adipose and muscle cells, by induction of a GPI-specific phospholipase C (GPI-PLC), as well as to stimulate glucose transport and utilization, such as lipid and glycogen synthesis, respectively [22,23,25,26,27,49,50,51,52] (for the concentration-dependent stimulation of lipid synthesis in human adipocytes by insulin and glimepiride, see also Supplementary Materials, Supplementary Figure S4a,b). From these data it might be concluded that both insulin and SUs interfere with the transfer of full-length GPI-APs from donor to acceptor cells, and the accompanying induction of anabolic processes and vice versa that the anabolic processes elicited by insulin and SUs are not mediated by intercellular GPI-AP transfer.

**Figure 3:**
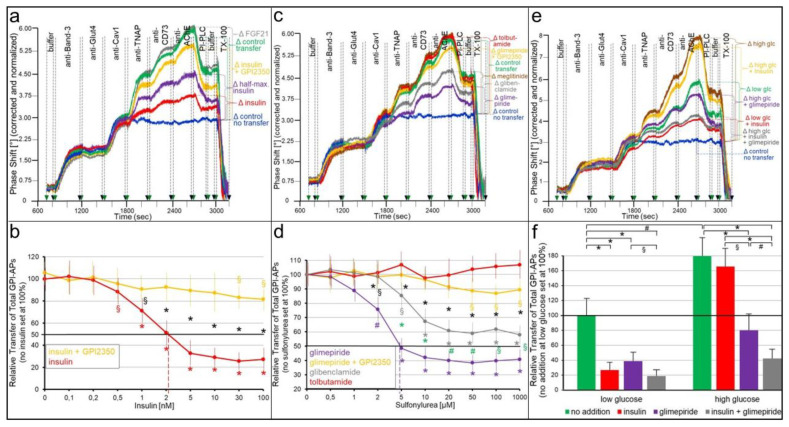
Inhibition of GPI-AP transfer from human adipocytes to ELCs by insulin and antidiabetic SUs. (**a**,**c**,**e**) Transwell co-cultures were run with human donor adipocytes (of lipid-loading stage II) or only medium (control no transfer) and GPI-deficient EL acceptor cells in the insert and bottom wells, respectively, as described in Section 4. After incubation (1 week, 37 °C) in the absence (control transfer) or presence of (**a**) human insulin (30 nM or 2 nM for half-max.) without or with the GPI-PLC inhibitor GPI2350 (100 μM) or of human FGF21 (50 nM), (**c**) tolbutamide (1 mM), glibenclamide (20 μM) or glimepiride (20 μM) without or with GPI2350 (100 μM) or of meglitinide (50 μM) and (**e**) glucose (5 mM for low glc; 20 mM for high glc) without or with insulin (30 nM) and/or glimepiride (20 μM), PMs were prepared from the ELCs of the bottom wells, coupled to chips by ionic/covalent capture and then analyzed for the expression of membrane proteins by SAW sensing, as described for Figure 1. Phase shifts induced by the binding of antibodies against GPI-APs or transmembrane proteins and by PI-PLC and TX-100 treatments (700–3200 s) are shown only, omitting the preceding capturing of PMs (0–700 s). The correction and normalization of the phase shift were performed as described for Figure 1. Phase shift Δ between the injection of the first (at 1800 s) and last antibody against GPI-APs (at 2700 s) are indicated by the horizontal, hatched lines and brackets for the various incubations. (**b**,**d**) The experiments (see **a**,**c**) were repeated three to six times with incubations at increasing concentrations of human insulin (**b**) or SUs (**d**) in the absence or presence of GPI2350 (100 μM). Phase shift Δ induced by antibodies against TNAP, CD73 and AChE (1800–2700 s) were corrected for those with medium alone in the insert wells (control no transfer) and used for the calculation of the relative transfer of total GPI-APs with the absence of insulin and SUs, respectively, set at 100% each. Significant differences between the absence and presence of insulin (**b**) or SUs (**d**) at the various concentrations, as well as between the absence and presence of GPI2350 for incubations with insulin (**b**) or glimepiride (**d**) at each concentration and between glimepiride and glibenclamide at each concentration (**d**) are indicated with the black and green symbols (**b**,**d**) and correspondingly colored symbols (**d**), respectively. (**f**) The experiments (see e) were repeated four or six times (distinct co-cultures) for incubations at low (0.1 mM) or high (5 mM) glucose in the absence (green bars) or presence of insulin (30 nM; red bars), glimepiride (20 μM; turquoise) or insulin together with glimepiride (grey bars). Phase shift Δ induced by antibodies against TNAP, CD73 and AChE (1800–2700 s) were corrected for those with medium alone in the insert wells (absence of insulin and SUs) and used for the calculation of the relative transfer of total GPI-APs (no addition at low glucose set at 100%). Significant differences between the relative transfer of total GPI-APs at low or high glucose at each addition are indicated (means ± SD; * *p* ≤ 0.01, # *p* ≤ 0.02, ^§^ *p* ≤ 0.05).

**Figure 4:**
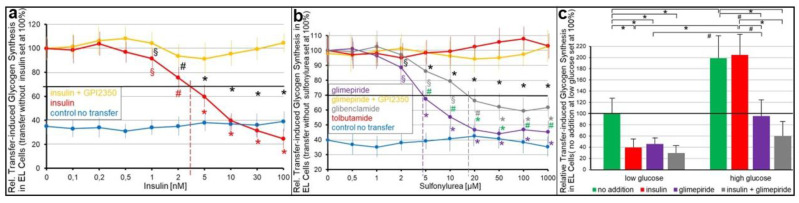
Inhibition of transfer-induced glycogen synthesis in ELCs by insulin and antidiabetic SUs. Transwell co-cultures were run with human donor adipocytes (of lipid-loading stage II) or only medium ((**a**,**b**); control no transfer) and GPI-deficient EL acceptor cells in the insert and bottom wells, respectively, as described in Section 4. After incubation (1 week, 37 °C) in the absence or presence of increasing concentrations of human insulin (**a**) or glimepiride, glibenclamide and tolbutamide (**b**) without or with GPI-PLC inhibitor GPI2350 at 100 μM (**a**,**b**) or (**c**) in the presence of low (0.1 mM) or high (5 mM) glucose without or with insulin (30 nM) or glimepiride (20 μM) or insulin together with glimepiride as indicated, the ELCs in the bottom plate were assayed for glycogen synthesis (15 min, 37 °C, presence of BSA, 0.1 mM [U-^14^C]glucose) as described in Section 4. The experiments were repeated four to six times (distinct co-cultures) with the determination of glycogen synthesis in triplicate. The relative transfer-induced glycogen synthesis in ELCs is given, with the absence of insulin (**a**), SUs (**b**) or both insulin and glimepiride at low glucose (**c**) set at 100%. Significant differences between the absence and presence of insulin (**a**) or SUs (**b**) at the various concentrations each are indicated with correspondingly colored symbols. Significant differences between the absence and presence of GPI2350 for incubations at each concentration of insulin (**a**) or glimepiride (**b**) and between glimepiride and glibenclamide at each concentration (**b**) are indicated with black and green symbols, respectively. (**c**) Significant differences between the various incubations at either low (left panel) or high glucose (right panel), as well as between low and high glucose in the absence or presence of insulin or glimepiride, are indicated (means ± SD; * *p* ≤ 0.01, # *p* ≤ 0.02, ^§^ *p* ≤ 0.05).

In fact, the presence of insulin (Figure 3a,b) or SUs (Figure 3c,d) during transwell co-culture led to the concentration-dependent reduction of the transfer of TNAP, CD73 and AChE from human adipocytes to GPI-deficient ELCs by up to 77% (30 nM insulin) and 61% (50 μM glimepiride) with the half-maximal inhibition at 2.3 nM and 4.9 μM, respectively. Importantly, FGF21 (Figure 3a), which exerts anabolic effects in adipocytes [53], as well as the insulin-releasing drugs meglitinide [54] and tolbutamide [55] (Figure 4c,d), had no effect on the transfer. Both insulin and SU inhibition of transfer was almost completely abrogated by an inhibitor of the GPI-PLC, GPI2350 [52] (Figure 3a–d). This argued for the lipolytic cleavage of the GPI-APs upon exposure of the donor human adipocytes to insulin, glimepiride and glibenclamide prior to initiation and completion of their transfer, in that ranking order of decreasing potency, as reflected in the leftward shift of the concentration–response curve of the former vs. the latter (Figure 3d).

Remarkably, the transfer of each GPI-AP studied was significantly elevated by glucose at high (5 mM) compared to low (0.1 mM) concentration (as routinely used in the transwell co-culture) (Figure 3e,f). This may be explained best by the upregulation of release from rather than insertion into the PMs of human adipocyte donor and EL acceptor cells, respectively. Previous findings have shown that release rather than insertion and donor rather than acceptor cells are susceptible towards stimulatory exogenous and endogenous factors, such as hyperglycemia or hyperinsulinemia [56], age or lipid-loading [33]. Strikingly, at high glucose, insulin failed to block total GPI-AP transfer (Figure 3e,f). At variance, the inhibition of transfer by glimepiride alone or together with insulin was maintained at high glucose concentrations (Figure 3e,f) and only tended to be lower compared to low glucose (55.6 vs. 61.3% alone and 76.9 vs. 80.7% with insulin). Apparently, at high glucose, glimepiride but not insulin manages to compensate for the increased glucose-dependent release of full-length GPI-APs by the activation of the GPI-PLC and thereby to restrict transfer. This can be explained best by the reported failure of high glucose to elicit the desensitization of the GPI-PLC towards stimulation by insulin but not by glimepiride [57].

To assess whether the inhibition of intercellular GPI-AP transfer by insulin and SUs leads to impairment of transfer-induced glycogen synthesis, the acceptor ELCs from the above transwell co-culturing with donor adipocytes (see Figure 3) were incubated with [U-^14^C]glucose after the termination of transfer (i.e., removal of the adipocytes). Insulin (Figure 4a), as well as glimepiride (Figure 4b), did not affect the glycogen synthesis in ELCs that had been cultured only with medium (control no transfer). This is in agreement with the complete lack of responsiveness of those cells towards both insulin and glimepiride (see Supplementary Materials, Supplementary Figure S4c,d).

The co-culturing of human adipocytes and ELCs in the absence of insulin and SUs led to 2.5- to 3.1-fold increases in glycogen synthesis (Figure 4a,b) due to the GPI-AP transfer, in agreement with previous findings [30]. Both insulin (Figure 4a) and glimepiride (Figure 4b) caused the downregulation of transfer-induced glycogen synthesis in a concentration-dependent fashion with IC_50_ of 2.8 nM and 4.3 μM, respectively. Glibenclamide (IC_50_ of 19.1 μM) was significantly less potent, and tolbutamide was inactive (Figure 4b). As expected, insulin (Figure 4a), as well as glimepiride (Figure 4b), inhibition of transfer-induced glycogen synthesis was abrogated by GPI2350, compatible with the inhibition of insulin- and glimepiride-induced cleavage and transfer of GPI-APs.

Strikingly, glucose at high concentrations present during transfer significantly stimulated transfer-induced glycogen synthesis in ELCs (Figure 4c, right panel), which was not abrogated by insulin (at variance with low glucose, see left panel). In contrast, it was impaired by glimepiride alone or in combination with insulin. The reduction by approximately 60 and 70%, respectively (Figure 4c, right panel), was of a similar degree as measured for low glucose (see left panel). Together, these findings imply that both insulin and SUs stimulate the lipolytic release of GPI-APs from the human adipocytes and thereby prevent their transfer to and induction of transfer-induced glycogen synthesis in the ELCs. 

### 2.3. Insulin and SU inhibition of GPI-AP Transfer and Transfer-Induced Glycogen Synthesis Is Controlled by Serum

Apparently, the inhibition of GPI-AP transfer to and transfer-induced glycogen synthesis in acceptor cells is provoked by three structurally different signals, insulin and antidiabetic SUs of the 2nd and 3rd generation, as demonstrated above, and certain serum proteins, such as GPLD1 and BSA, as has been shown previously [30], and based on two different molecular mechanisms of interference with GPI-AP transfer, cleavage by insulin-/SU-dependent GPI-PLC (see above) and interaction with certain serum GPI-binding proteins [30]. This raised the possibility of the sub-additive, additive or synergistic inhibition of GPI-AP transfer, as well as transfer-induced glycogen synthesis, in the course of cooperation of insulin or SUs and serum.

**Figure 5:**
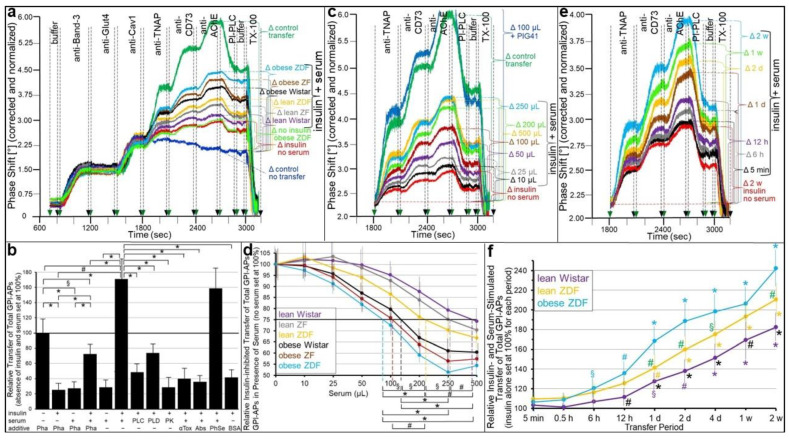
Restoration of insulin-inhibited GPI-AP transfer by serum. (**a**–**f**) Transwell co-cultures were run with human donor adipocytes (of lipid-loading stage II) or only medium (only a; control no transfer) and GPI-deficient EL acceptor cells in the insert and bottom wells, respectively, as described in Section 4. The co-cultures were incubated (37 °C, 5 mM glucose) ((**a**–**d**), 1 week; (**e**,**f**), increasing periods of time) in the absence (only (**a**–**c**); control transfer) or presence of human insulin (30 nM, (**a**–**f**)) without (**a**–**c**,**e**) or with serum (**a**,**b**,**e**,**f**, 100 µL; **c**,**d**, increasing volumes; see brackets), which was prepared from lean or obese Wistar, ZF or ZDF rats (**a**,**d**,**f**) or obese ZDF rats (**b**,**c**,**e**) and then diluted 10-fold with PBS containing 2 mM Pha in the presence of Ca^2+^ (4 mM) (**b**), Pha (1 mM) ((**a**–**f**,**b**) as indicated) or PIG41 (100 µM) (**c**). Thereafter PMs were prepared from the ELCs of the bottom wells, coupled to chips by ionic/covalent capture and then analyzed for the expression of membrane proteins by SAW sensing, as described for Figure 1. Phase shifts induced by the binding of antibodies against TMPs (800–1800 s; only shown for (**a**)) and GPI-APs (1800–2700 s) and by subsequent PI-PLC and TX-100 treatments (2700–3200 s) are shown only, omitting the preceding capturing of the PMs (0–700 s). The serum was left untreated (**a**–**f**) or (**b**) digested with bacterial PI-PLC (PLC), human GPLD1 (PLD) or proteinase K (PK) or supplemented with α-toxin (αTox) or antibodies against TNAP, CD73 and AChE (Abs), each coupled to Sepharose beads or phenyl Sepharose beads (PhSe), as described in Section 4. In addition, BSA (4 mg/mL PBS) was added instead of serum (**b**). (**a**,**c**,**e**) Correction and normalization of the phase shift were performed as described for Figure 1. Phase shift Δ between the start of injection of anti-TNAP antibody (at 1800 s) and termination of the injection of anti-AChE antibody (at 2700 s) are indicated by the horizontal, hatched lines and brackets for each incubation condition. (**b**) The experiment (see (**a**)) was repeated three to five times (distinct co-cultures) and SAW sensing in quadruplicate. Phase shift Δ induced by antibodies against TNAP, CD73 and AChE (1800–2700 s) were corrected for medium alone in the insert wells (see (**a**); control no transfer) and used for the calculation of the relative transfer of total GPI-APs in the absence of both insulin and serum (see (**a**); control transfer) set at 100%, with biologically relevant significant differences indicated. (**c**,**d**) The experiment (see (**b**)) was repeated six times (distinct co-cultures) and SAW sensing in triplicate. Phase shift Δ induced by antibodies against TNAP, CD73 and AChE (1800–2700 s) were corrected for medium alone in the insert wells (**c**) and used for the calculation of the relative insulin-inhibited transfer of total GPI-APs in presence of serum (**d**), with differences between the control transfer and transfer left in the presence of insulin and absence of serum set at 100%. Significant differences in the volumes (IV_50_) required for a 50% reduction of the maximal insulin-inhibited transfer (horizontal, black line) between the different types of serum are indicated (vertical, colored, and hatched lines). (**f**) The experiment (see (**e**)) was repeated three to six times (distinct co-cultures and SAW sensing in quadruplicate) without and with serum from rats of different metabolic states in the presence of insulin (30 nM) for increasing transfer periods. Phase shift Δ induced by antibodies against TNAP, CD73 and AChE (1800–2700 s) were corrected for medium alone in the insert wells and used for the calculation of the relative insulin- and serum-stimulated transfer of total GPI-APs, with the transfer left after two weeks in the presence of insulin and absence of serum (see (**e**)) set at 100% for each transfer period. Significant differences vs. 5 min transfer period are indicated for each type of serum by the correspondingly colored symbols, as well as between obese ZDF and lean ZDF or lean Wistar rats by green or black symbols, respectively (means ± SD; * *p* ≤ 0.01, # *p* ≤ 0.02, ^§^ *p* ≤ 0.05).

As expected, both insulin alone (Figure 5a,b; no serum) and serum (from obese ZDF rats) alone without (Figure 5b) or with the Ca^2+^-chelating agent Pha (Figure 5a,b; no insulin) decreased the transfer of the GPI-APs studied (Figure 5a; control transfer) by approximately 75% compared to the absence of donor cells (Figure 5a; control no transfer). This indicated the efficient reduction of the number of GPI-APs competent for transfer in the course of insulin-induced cleavage or binding to serum proteins, respectively, of the GPI anchors. Importantly, the serum effects cannot be attributed to insulin, which was certainly left in the serum samples at considerable concentrations, in particular, in those obtained from obese rats (see Supplementary Materials, Supplementary Table S2). Firstly, the samples were considerably diluted resulting in a final assay concentration of rat insulin, which fell below the insulin sensitivity of even typical insulin target cells, such as adipocytes. Secondly, the ELCs did not display any insulin responsiveness (see Supplementary Materials, Supplementary Figure S4c) due to the fact of the missing expression of insulin receptors [39]. However, unexpectedly, the combination of insulin and serum (from obese ZDF rats) in the presence of Pha led to the restoration of the transfer to approximately 70% (Figure 5a,b) compared to the maximal transfer in the absence of both (Figure 5a, control transfer; b). Strikingly, in the absence of Pha, the transfer was even further increased to approximately 170% (Figure 5b). The latter indicates that Ca^2+^ fosters the stimulation of transfer by the combined action of insulin and serum. This may be due to the weakening of the interaction between certain serum GPI-binding proteins, such as GPLD1, and full-length GPI-APs, which has previously been shown to depend on Ca^2+^ [34].

An explanation for the apparent contradiction that insulin and serum each, per se, inhibit but in concert stimulate GPI-AP transfer may be that serum GPI-binding proteins, such as GPLD1 and BSA, act as source of full-length GPI-APs, from which they become transferred upon their release in response to insulin. This hypothesis was tested by various pretreatments of serum (from obese ZDF rats) envisaged for the depletion of full-length GPI-APs (Figure 5b). In fact, the digestion of serum with bacterial PI-PLC, human GPLD1 and proteinase K, which cleave off the GPI anchor and protein moiety, respectively, of the GPI-APs, as well as the addition of α-toxin or anti-TNAP/CD73/AChE antibodies, each coupled to Sepharose beads, which bind to the GPI anchor of each and the protein moiety of individual GPI-APs, respectively, significantly reduced the concerted insulin- and serum-stimulated transfer of each GPI-AP at varying degrees. Interestingly, pretreatment of serum with phenyl Sepharose beads, which has previously been demonstrated to bind to the anchor of detergent-solubilized GPI-APs [58], did not affect the transfer of GPI-APs (Figure 5b). This is presumably due to the fact of their tight interaction with serum GPI-binding proteins, which prevents binding of phenyl Sepharose to the GPI anchors and thereby interference of the beads with insertion of the anchors into the PMs of acceptor cells (Figure 5b).

BSA has previously been shown to interact with full-length GPI-APs [30]. Nevertheless, defatted BSA from commercial sources did not substitute for serum in supporting insulin stimulation of transfer (Figure 5b). This is explained best by those charges of BSA having lost full-length GPI-APs during the defatting procedures. Together, these data supported the view that serum (from obese ZDF rats) provides full-length GPI-APs, which are bound to proteins and apparently released thereof in response to insulin, thereby gaining competence for transfer to acceptor cells.

In accordance with this explanation, the restoration of the insulin-inhibited GPI-AP transfer (Figure 5c; insulin no serum) by serum (from obese ZDF rats) (Figure 5c) was dependent on its volume and reached 50% of the control transfer (absence of insulin and serum) with 250 µL (Figure 5c,d). Moreover, it was affected by the nature of the serum (Figure 5a,d), with that from obese ZDF rats being most effective (Figure 5a; to up to 70% of control transfer), followed by serum from obese ZF, obese Wistar, lean ZDF, lean ZF and lean Wistar rats in that ranking order of declining efficacy (Figure 5a). This ranking order was also reflected in the volumes of the different sera required for the half-maximal restoration of insulin-inhibited transfer (Figure 5d). In fact, the calculation of the IV_50_ enabled the differentiation between the different metabolic states, except for obese ZDF and obese ZF rats, as well as obese ZF and obese Wistar rats (Figure 5d). Metabolically dysregulated rats were chosen since they represent a widely acknowledged animal model of type II diabetes mimicking the different stages of normoglycemia/insulinemia- to hyperglycemia/insulinemia (see Supplementary Materials, Supplementary Table S2 for a detailed characterization of the metabolic states) along the pathogenesis, driven by both genotype (Wistar, ZF and ZDF) and feeding (normal and high-fat diet) (for a review, see [59,60,61]).

The above conclusion that full-length GPI-APs bound to certain serum proteins become transferred to acceptor ELCs in the course of action of insulin-dependent GPI-PLC at the donor adipocytes raised the question concerning the underlying mechanism. Strikingly, PIG41, which structurally closely resembles the glycan core of human AChE [62,63], in combination with serum (100 μL, obese ZDF) led to drastically increased transfer (Figure 5c) compared to insulin combined with serum, which even exceeded the control transfer (absence of serum and insulin). This suggests that PIG41 causes dissociation of full-length GPI-APs from serum GPI-binding proteins. Thus, in the transwell co-culture in the presence of serum, the rate of the intercellular transfer of GPI-APs was determined by the efficacy of their dissociation from GPI-binding proteins and their amounts, i.e., serum volume and type. This agrees with previous [30,34] and the above findings (Figure 5b) that only full-length GPI-APs not bound to serum proteins are competent for transfer.

The transfer in response to the combination of insulin and serum was time dependent (Figure 5e,f) and detectable after 5 min at the earliest and then for up to two weeks. The combined insulin- and serum-stimulated transfer (with insulin inhibition for each period subtracted) was positively correlated to the transfer period (Figure 5f) and dependent on the type of serum. Serum from obese ZDF and lean Wistar rats was most and least potent, respectively (Figure 5f). These findings add further evidence that in the presence of insulin and serum in the transwell co-culture, the full-length GPI-APs that were competent for transfer most likely originated from serum GPI-binding proteins rather than the donor adipocytes.

**Figure 6:**
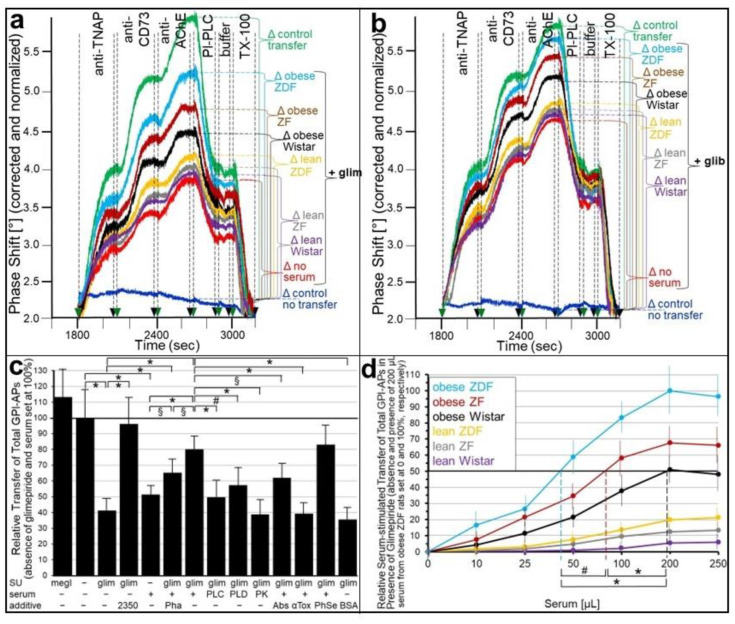
Abrogation of the SU inhibition of GPI-AP transfer by serum. (**a**–**d**) Transwell co-cultures were run with human donor adipocytes (of lipid-loading stage II) or only medium (shown only for (**a**,**b**); control no transfer) and GPI-deficient EL acceptor cells in the insert and bottom wells, respectively, as described in Section 4. The co-cultures were incubated (1 week, 37 °C, 5 mM glucose) in the absence ((**a**,**b**); control transfer) or presence of 30 µM glimepiride (**a**,**d**) without or with 100 µM GPI2350 (c; 2350) or 30 µM glibenclamide (**b**) or 50 µM meglitinide (c; megl) in the absence ((**a**,**b**); no serum) or presence of 100 µL (**a**–**c**) or increasing volumes (**d**) of serum, which had been prepared from lean or obese Wistar, ZF or ZDF rats (**a**,**b**,**d**) or obese ZDF rats (**c**) and then diluted 10-fold with PBS containing 2 mM Pha, or of 100 µL of BSA (4 mg/mL PBS) in the presence of 4 mM Ca^2+^ (**a**–**d**) or 1 mM Pha (only (**c**)). PMs were prepared from the ELCs of the bottom wells, coupled to chips by ionic/covalent capture and then analyzed for the expression of GPI-APs by SAW sensing, as described for Figure 1. Phase shifts induced by the binding of antibodies against GPI-APs and subsequent treatments with PI-PLC and TX-100 (1800–3200 s) are shown only, omitting the preceding capturing of PMs (0–700 s), as well as the binding of antibodies against TMPs (700–1800 s). Serum samples were left untreated (**a**,**b**,**d**) or (**c**) digested with bacterial PI-PLC (PLC), human GPLD1 (PLD) or proteinase K (PK) or supplemented with antibodies against TNAP, CD73 and AChE (Abs) or α-toxin (αTox), each coupled to Sepharose beads or phenyl Sepharose beads (PhSe) or Pha (1 mM) prior to addition to the transwell co-cultures. (**a**,**b**) Correction and normalization of the phase shift were performed, as described for Figure 1. Phase shift Δ between the start of the injection of the anti-TNAP antibody (at 1800 s) and termination of the injection of anti-AChE antibody (at 2700 s) are indicated by horizontal, hatched lines and brackets for each incubation condition. (**c**) The experiment was repeated four times (distinct co-cultures with SAW sensing in triplicate). Phase shift Δ induced by antibodies against TNAP, CD73 and AChE (1800–2700 s) were corrected for medium alone in the insert wells and used for the calculation of the relative transfer of total GPI-APs, with the absence of SUs, serum and additives set at 100% (horizontal, black line). Biologically relevant significant differences are indicated. (**d**) The experiment (see (**a**)) was repeated three to five times (distinct co-cultures with SAW sensing in duplicate) with increasing volumes of sera from rats of different metabolic states in the presence of 30 µM glimepiride. Phase shift Δ induced by antibodies against TNAP, CD73 and AChE (1800–2700 s) were corrected for medium alone in the insert wells and used for the calculation of the relative serum-stimulated transfer of total GPI-APs in the presence of glimepiride with the absence and presence of 200 µL serum from obese ZDF rats set at 0 and 100%, respectively. Significant differences between the volumes (EV_50_) effective in the 50% stimulation of glimepiride-inhibited transfer (horizontal, black line) of sera from obese Wistar, ZF and ZDF rats (vertical, colored, and hatched lines) are indicated (means ± SD; * *p* ≤ 0.01, # *p* ≤ 0.02, ^§^ *p* ≤ 0.05).

The transfer of full-length GPI-APs upon their dissociation from serum GPI-binding proteins by either PIG41 or insulin suggests that lipolytically cleaved GPI-APs (PIG-proteins) released from donor cells by the insulin-/SU-dependent GPI-PLC mediate GPI-AP transfer from human adipocytes to ELCs during the simultaneous presence of serum and SUs. As expected, glimepiride or glibenclamide alone (Figure 6a,b; no serum), as well as serum from obese ZDF rats (without or with Pha; Müller and Müller, unpublished data) alone (Figure 6c), reduced the transfer by approximately 50% compared to their absence (Figure 6a,b; control transfer, 6c). The SU inhibition was almost fully abrogated by the GPLD1 inhibitor GPI2350 (Figure 6c; shown only for glimepiride). This confirmed the prevention of GPI-APs from transfer by SUs as a consequence of their lipolytic cleavage or by serum GPI-binding proteins in the course of binding to their GPI anchors.

However, the combination of glimepiride or glibenclamide and serum (from obese ZDF rats) led to approximately 80 and 90% transfer, respectively, of that in absence of both SU and serum (Figure 6a,b; control transfer, 6c). The presence of Pha significantly reduced the combined serum- and glimepiride-induced transfer (Figure 6c). This was compatible with the above explanation that the absence of Ca^2+^ interferes with transfer as a result of the stabilization of the interaction between full-length GPI-APs and serum GPI-binding proteins.

The possibility that serum GPI-binding proteins act as a source for full-length GPI-APs that are competent for transfer upon their SU-induced dissociation was investigated. The digestion of serum (from obese ZDF rats) with bacterial PI-PLC, human GPLD1 or proteinase K, as well as the addition of combined anti-TNAP/CD73/AChE antibodies or α-toxin coupled to Sepharose beads, significantly impaired the combined SU- and serum-induced transfer at variable degrees compared to the untreated serum (Figure 6c). Only phenyl Sepharose beads had no effect (see Figure 5b). BSA instead of serum did not prevent glimepiride inhibition of transfer. In conclusion, the data were compatible with full-length GPI-APs bound to serum GPI-binding proteins becoming competent for transfer upon their dissociation in response to the glimepiride challenge of donor cells.

The abrogation of the SU inhibition of transfer by serum was dependent on the metabolic state of the donor rats (Figure 6a,b) and the volume used (Figure 6d). Serum from obese ZDF rats turned out to be the most effective for both glimepiride (Figure 6a) and glibenclamide (Figure 6b) inhibition compared to the control transfer (absence of both SU and serum), followed by obese ZF, obese Wistar, lean ZDF, lean ZF and lean Wistar rats in that ranking order of declining efficacy. This ranking order was also reflected in the volumes of the different sera effective in the half-maximal stimulation of transfer (EV_50_) in the presence of glimepiride (Figure 6d), which enabled the differentiation between obese ZDF and ZF, ZF and Wistar or ZDF and Wistar rats.

**Figure 7:**
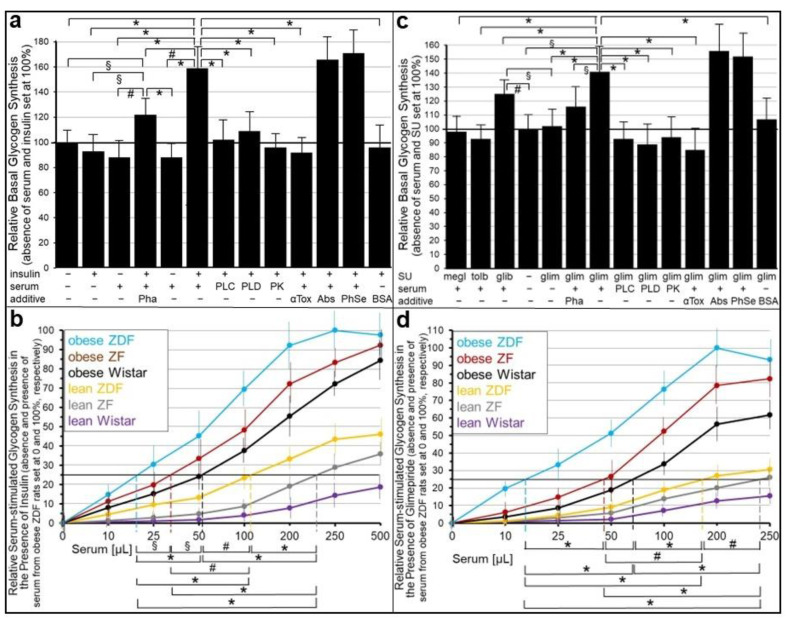
Stimulation of glycogen synthesis in ELCs by GPI-AP transfer in the simultaneous presence of serum and either insulin or SUs. (**a**,**c**) Human donor adipocytes (of lipid-loading stage II) were incubated (1 week, 37 °C, 5 mM glucose) with GPI-deficient EL acceptor cells in the insert and bottom wells, respectively, of transwell co-cultures in the absence or presence of human insulin (30 nM), meglitinide (megl, 50 μM), tolbutamide (tolb, 1 mM), glibenclamide (glib, 30 μM) and glimepiride (glim, 30 μM) without or with 200 µL serum, which were prepared from obese ZDF rats and then diluted 10-fold with PBS containing 2 mM Pha, or BSA (4 mg/mL PBS) in the presence of 4 mM Ca^2+^ or 1 mM Pha, as indicated (**a**,**c**). The serum was left untreated or digested with bacterial PI-PLC (PLC), human GPLD1 (PLD) or proteinase K (PK) or supplemented with α-toxin (αTox) or antibodies against TNAP, CD73 and AChE (Abs), each coupled to Sepharose beads or phenyl Sepharose beads (PhSe) prior to addition to the transwell co-cultures. (**b**,**d**) The transwell co-cultures were run as above without or with increasing volumes of untreated serum, which were prepared from lean or obese Wistar, ZF or ZDF rats under the inclusion of Pha (2 mM), in the presence of insulin (30 nM) (**b**) or glimepiride (30 μM) (**d**). Thereafter, the ELCs in the bottom plate were assayed for glycogen synthesis (15 min, 37 °C, presence of BSA, 0.1 mM [U-^14^C]glucose), as described in Section 4. The transwell co-cultures were repeated three to five times (distinct co-cultures) with determination of glycogen synthesis in triplicate. (**a**,**c**) The relative glycogen synthesis is given with the absence of serum and insulin (**a**) or SUs (**c**) set at 100% (horizontal, black lines). Significant differences are indicated. (**b**,**d**) The relative serum-stimulated glycogen synthesis in the presence of either insulin (**b**) or glimepiride is given, with the absence and presence of serum (250 and 200 μL, respectively) from obese ZDF rats set at 0 and 100%, respectively. Serum volumes (EV_25_) effective in the 25% stimulation of glycogen synthesis (horizontal, black lines) in the presence of either insulin (**b**) or glimepiride (**d**) are indicated by the vertical, hatched, and colored lines. Significant differences between EV_25_ for sera from rats of different metabolic states are indicated (means ± SD; * *p* ≤ 0.01, # *p* ≤ 0.02, ^§^ *p* ≤ 0.05).

Next it was investigated whether the abrogation of the insulin and SU inhibition of GPI-AP transfer by serum is reflected in the stimulation of glycogen synthesis in the acceptor cells. For this, human adipocytes as donor cells and GPI-deficient ELCs as acceptor cells were incubated in transwell co-culture in the presence of insulin (Figure 7a,b) or SUs (Figure 7c,d) without or with serum from rats of different metabolic states (Figure 7b,d), which had been pretreated to get rid of (full-length) GPI-APs (Figure 7a,c). The assaying of the ELCs for basal glycogen synthesis (5 mM glucose) revealed that serum (from obese ZDF rats), insulin and glimepiride each alone and independent of the absence or presence of Pha (Müller and Müller, unpublished results) did not exert any significant effect (Figure 7a,c). However, either insulin or glimepiride or glibenclamide but not tolbutamide or meglitinide in combination with serum significantly stimulated glycogen synthesis in that ranking order of declining potency. These effects were considerably diminished by Pha. Lipolytic and proteolytic pretreatments of the serum, as well as α-toxin Sepharose beads, completely blocked serum-stimulated glycogen synthesis in the presence of either insulin (Figure 7a) or glimepiride (Figure 7c). Strikingly, antibodies against TNAP, CD73 and AChE coupled to Sepharose beads had no impact. BSA did not substitute for serum in stimulating glycogen synthesis (Figure 7a,c). Thus, the positive correlation between the upregulation of GPI-AP transfer (see Figure 5 and Figure 6) and glycogen synthesis in ELCs (see Figure 7a,c) in the course of the combined challenge of donor cells with serum and either insulin or glimepiride is compatible with full-length GPI-APs, which are not identical with TNAP, CD73 and AChE, mediating the upregulation of basal glycogen synthesis upon their dissociation from serum GPI-binding proteins, which are not identical with albumin, and subsequent transfer to the PMs of the acceptor cells.

Subsequent analysis of the volume dependence for sera from rats of different metabolic state in stimulating glycogen synthesis in acceptor ELCs in the presence of either insulin (Figure 7b) or glimepiride (Figure 7d) revealed identical rankings, i.e., obese ZDF, obese ZF, obese Wistar, lean ZDF, lean ZF, lean Wistar, in that order of decreasing potency, which were identical to those for serum stimulation of GPI-AP transfer in the presence of insulin (Figure 5) or glimepiride (Figure 6).

### 2.4. Full-Length GPI-APs Displaced from Serum Proteins by PIG(-Proteins) Are Transferred to and Stimulate Glycogen Synthesis in Acceptor Cells

**Figure 8:**
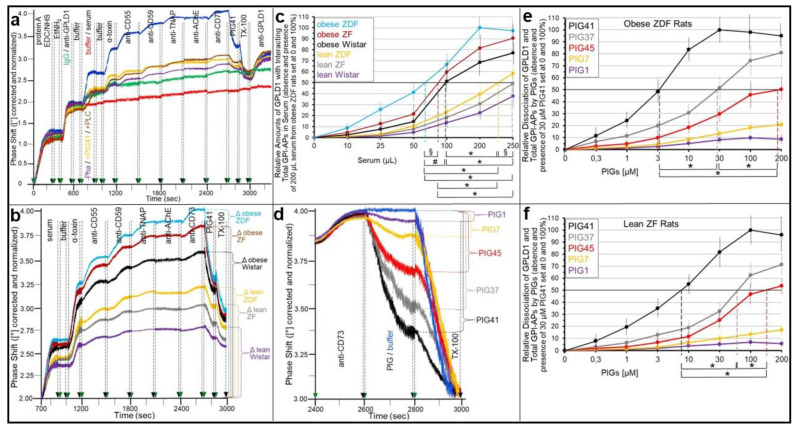
Binding to and displacement by PIGs from GPLD1 of full-length GPI-APs of serum from metabolically dysregulated rats. (**a**) After the covalent immobilization of protein A onto EDC/NHS-activated chips (0–300 s) and subsequent blockade of unreacted carboxyl groups by ethanolamine (EtNH_2_) (300–400 s), monoclonal anti-GPLD1 antibody (blue, yellow, brown and turquoise curves) or IgG (green curve) was injected into the chip channels. Following the washing of the chips (600–700 s), serum (200 μL, prepared from obese ZDF rats and then diluted 10-fold with PBS containing 2 mM Pha), which was pretreated with bacterial PI-PLC (0.2 mU/mL, brown curve) or remained untreated (blue, yellow, turquoise and green curves), or buffer (red curve) was injected (700–900 s) at a flow rate of 60 μL/min in the absence (turquoise curve) or presence of 1 mM Pha (all other curves) or 30 µM PIG41 (yellow curve). After washing of the channels with buffer (900–1000 s) at a flow rate of 200 μL/min and then with α-toxin (30 μg/mL, 1000–1200 s), anti-CD55 (1200–1500 s), CD59 (1500–1800 s), TNAP (1800–2100 s), AChE (2100–2400 s) and CD73 (2400–2700 s) antibodies (at the dilutions given in Supplementary Materials, Materials) were injected successively at a flow rate of 15 μL/min. After the injection of 30 μM PIG41 (2700–2850 s) together with 4 mM Ca^2+^ and then 0.2% TX-100 (2850–3000 s) at a flow rate of 45 μL/min, polyclonal anti-GPLD1 antibody was finally injected (3000–3300 s) at a flow rate of 15 μL/min. The experiment was repeated two times (distinct chips) with similar results (representative shown). Phase shift is given upon correction for unspecific interaction of serum components (“mock” channel lacking protein A) and altered viscosity (vs. buffer) of the sample fluid and normalization for the varying efficiencies of distinct chips for capture of protein A. (**b**) The experiment was performed as described (see (**a**), blue curve; chips with immobilized anti-GPLD1, absence of PIG41) using untreated sera prepared from rats of different metabolic states and diluted with PBS containing 2 mM Pha) as indicated and repeated four to six times (distinct chips with four injections per incubation) with similar results (representative shown). The measured phase shift was corrected (see (**a**)) and is only shown from the start of serum (700 s) to the end of TX-100 injection (3000 s). Phase shift increases induced by serum, α-toxin and antibodies (700–2700 s) are indicated for each type of serum (horizontal, hatched lines and brackets). (**c**) The experiment was repeated (see (**b**)) three to five times (distinct chips with four to six injections per incubation) with increasing volumes (i.e., adjusting the flow rate from 15 to 75 μL/min to cover 50–250 µL and from 30 to 75 μL/min to cover 10–25 μL) of the untreated sera (diluted 1:10 and 1:100 with PBS containing 2 mM Pha, respectively) from rats of different metabolic states as indicated. The relative amounts of GPLD1 with interacting GPI-APs, as reflected in the sum of the serum-, α-toxin- and antibody-induced phase shift increases (see (**b**); 700–2700 s), are given, with the absence (200 μL of buffer; see a, red curve) and presence of serum (200 μL from obese ZDF rats; see a,b, blue curves) set at 0 and 100%, respectively. Serum volumes (EV_50_) effective in the 50% increase in the relative amounts of GPLD1 interacting with GPI-APs (as shown by the horizontal, black line) are indicated by the vertical, hatched, and colored lines. Significant differences between EV_50_ of sera from rats of different metabolic states are indicated. (**d**) The experiment was performed as described (see (**a**); chips with immobilized anti-GPLD1) with untreated serum, prepared from obese ZDF rats and diluted with PBS containing Pha, and subsequent injection of PIG41, 37, 45, 7 and 1 (30 μM) or buffer together with Ca^2+^ (4 mM) (blue curve) and repeated once (distinct chips with four channels per incubation) with similar results (representative shown). The measured phase shift was corrected (see (**a**)) and is only shown from the start of the last antibody (2400 s) to the end of the TX-100 injection (3000 s). PIG-induced phase shift decreases (2600–2800 s) are indicated (horizontal, hatched lines and brackets). (**e**,**f**) The experiment was repeated (see (**d**)) four to six times (distinct chips with four to eight channels per incubation) using increasing concentrations of PIGs together with Ca^2+^ (4 mM) and with serum (200 μL) from obese ZDF (**e**) and lean ZF rats (**f**). The relative dissociation of GPLD1 and GPI-APs by PIGs, as reflected in the PIG-induced phase shifts (2600–2800 s), is given with the absence and presence of 30 (**e**) or 100 μM PIG41 (**f**) set at 0 and 100%, respectively. The concentrations of PIGs effective in the 50% increase in dissociation (EC_50_; shown by horizontal, black lines) are indicated by the vertical, hatched, and colored lines. Significant differences between EC_50_ of the different PIGs are indicated (means ± SD; * *p* ≤ 0.01, # *p* ≤ 0.02, ^§^ *p* ≤ 0.05).

The above experiments revealed that serum from obese ZDF rats added to the transwell co-culture abrogates insulin and SU inhibition of GPI-AP transfer (Figure 6), as well as glycogen synthesis in ELCs (Figure 7). These findings led to the hypothesis that full-length GPI-APs bound to serum GPI-binding proteins become displaced by PIG-proteins, lipolytically cleaved off from full-length GPI-APs of donor cells in response to insulin or SUs. Consequently, serum from rats of different metabolic state was assayed for binding of full-length GPI-APs and their displacement by PIGs or lipolytically cleaved GPI-APs (PIG-proteins). GPLD1 has been identified so far as the only serum protein that interacts with full-length GPI-APs [34]. Experimental demonstration of the interaction was favored by the presence of Pha, which inhibits the Ca^2+^-dependent lipolytic activity of GPLD1 [31,32]. Therefore, sera from rats of different metabolic states were studied for the expression of GPLD1 and its interaction with as well as displacement by PIG(-proteins) of GPI-APs (Figure 8).

For this, SAW chips were generated with protein A being covalently coupled to the gold surface and a monoclonal antibody against rodent GPLD1 subsequently being immobilized at their channels (Figure 8a). Both steps (0–400 and 400–700 s, respectively) were monitored by considerable increases in phase shift. The injection of serum from obese ZDF rats (700–900 s) together with Pha (Figure 8a; blue curve) but not without Pha (turquoise curve) caused the additional upregulation of phase shift vs. buffer (red curve) in the anti-GPLD1 antibody (blue curve) but not the anti-IgG control (green curve) channel. Phase shift was further elevated by the injection of α-toxin (1000–1200 s), as well as anti-CD55 (1200–1500 s), CD59 (1500–1800 s), TNAP (1800–2100 s) and CD73 (2400–2700 s), but not AChE antibodies (2100–2400 s) in a successive fashion (Figure 8a). This is compatible with binding to rather than cleavage by rat serum GPLD1 of GPI-APs in the absence of Ca^2+^, among them CD55, CD59, TNAP and CD73, which all represent minor constituents of rat serum [3,4,13].

**Figure 9:**
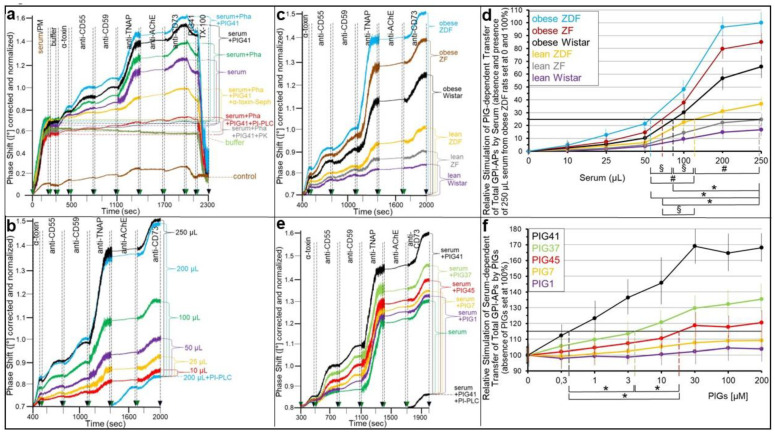
Transfer to ELCs of GPI-APs released from serum GPI-binding proteins of metabolically dysregulated rats by PIGs. (**a**–**f**) GPI-deficient EL acceptor cells were incubated (1 week, 37 °C, 5 mM glucose, 1 mM Ca^2+^) in the bottom wells of transwell co-cultures with 200 μL buffer (**a**) or serum (**a**,**c**,**e**,**f**, 200 µL; b,d, increasing volumes), which were prepared from obese ZDF rats (**a**,**b**,**e**,**f**) or rats of different metabolic states (**c**,**d**), then diluted 10-fold with PBS lacking ((**a**), as indicated) or containing 2 mM Pha (**a**–**f**) and thereafter left untreated ((**a**), as indicated; **b**–**f**) or incubated with proteinase K (PK), bacterial PI-PLC (PLC), α-toxin coupled to Sepharose beads (α-toxin) (**a**), 30 μM PIG41 (**a**–**d**) or different PIGs ((**e**), 30 µM; f, increasing concentrations). Thereafter, the ELCs were washed thoroughly by rinsing the bottom wells three times with 2 mL each of 20 mM Tris/HCl, 1.5 M NaCl and then two times with 2 mL each of PBS. Subsequently, PMs were prepared from the ELCs, coupled to chips by ionic/covalent capture (0–200 s), as described in Section 4, and then analyzed for expression of GPI-APs by SAW sensing. As a control, 200 μL serum from obese ZDF rats instead of PMs were injected into the chips (0–200 s) at a flow rate of 60 μL/min ((**a**) only, brown curve). After washing of the channels with buffer (200–300 s) at a flow rate of 200 μL/min, α-toxin (30 μg/mL, 300–500 s), anti-CD55 (500–800 s), CD59 (800–1100 s), TNAP (1100–1400 s), AChE (1400–1700 s) and CD73 (1700–1950 s) antibodies (at the dilutions given in Supplementary Materials, Materials) were injected successively at a flow rate of 15 μL/min, followed by 30 μM PIG41 (1950–2150 s, shown only for (**a**)) and then 0.2% TX-100 (2150–2300 s, shown only for (**a**)) at a flow rate of 45 μL/min. Measured phase shifts were corrected and normalized as described for Figure 1 (**a**) or by subtraction of the control (see (**a**), serum instead of PM, brown curve) values (**b**,**c**,**e**). (**a**,**c**) Phase shift Δ between the start of the α-toxin (300 s) and termination of the anti-CD73 antibody (1950 s) injection indicated by the horizontal, hatched lines ((**a**) only) are given by brackets. (**b**,**e**) Brackets indicate the difference between the total phase shift induced by α-toxin and all antibodies for each incubation condition and a control phase shift induced by pretreatment of 200 μL serum with PIG41 and bacterial PI-PLC (blue and black curves). (**d**,**f**) The experiments (see (**c**,**e**), respectively) were repeated (four or six incubations and distinct chips with four channels per incubation) using increasing volumes of untreated serum from rats of different metabolic states (**d**) or increasing concentrations of different PIGs (**f**). The relative stimulation of PIG- ((**d**), 30 μM PIG41) and serum- ((**f**), 250 μL, obese ZDF rats) dependent transfer of total GPI-APs, as reflected in the α-toxin- and antibody-induced phase shifts, are calculated with the absence and presence of 250 µL serum from obese ZDF rats set at 0 and 100%, respectively, (**d**) or absence of PIGs set at 100% (**f**). Significant differences between the volumes effective in the 25% stimulation (EV_25_, (**d**)) or concentrations effective in 15% stimulation (EC_15_, (**f**)) of transfer (horizontal black lines) by the different sera (**d**) and PIGs (**f**), respectively, are indicated (vertical, colored, and hatched lines) (means ± SD; * *p* ≤ 0.01, # *p* ≤ 0.02, ^§^ *p* ≤ 0.05).

The approximately 50% decreases of the α-toxin- and multiple-antibody-induced phase shifts following PIG41 and TX-100 injections (Figure 8a; blue curve) argued for the involvement of the GPI anchor glycan core and micelle-like complexes constituted by GPI-APs, cholesterol and (lyso)phospholipids [33,64], respectively, in the recognition of GPI-APs by GPLD1. The former hypothesis was confirmed by co-injection of PIG41 and serum or pretreatment of serum with bacterial PI-PLC (700–900 s), which both led to an approximately 50% reduction of the serum-, α-toxin- and antibody-induced phase shift increases (Figure 8a, yellow and brown curves, respectively). This hints to the critical role of the full-length GPI anchor for the interaction of GPLD1 and GPI-APs. Again, the remaining phase shift increase is explained best by only partial deacylation of the myo-inositol residue of the GPI anchor of the GPI-APs expressed in human ELCs, which is a prerequisite for their cleavage by bacterial PI-PLC (see above). The considerable increases in phase shift upon the final injection of a polyclonal antibody cross-reactive for human and rat GPLD1 in all channels, except for those with no GPLD1 or serum injected (Figure 9a; green and red curves, respectively), confirmed the capture of GPLD1 from the serum of obese ZDF rats. Taken together, SAW sensing using chips with immobilized anti-GPLD1 antibody can be used for the analysis of the interaction of full-length GPI-APs and GPLD1 in rat serum and their displacement by PIGs.

Under these conditions, the serum-, α-toxin- and anti-CD55-, CD59-, TNAP- and CD73-induced phase shift increases were most pronounced for serum from obese ZDF rats, followed by obese ZF, Wistar rats, lean ZDF, ZF rats and, lastly, lean Wistar rats (Figure 8b). The relative amounts of GPLD1 and interacting full-length GPI-APs contained in serum were determined with increasing serum volumes and the calculation of the EV_50_ for the half-maximal phase shift increases for each serum (Figure 8c). The lowest EV_50_ for obese ZDF rats was indicative of the highest amount of both serum GPLD1 and interacting full-length GPI-APs, including CD55, CD59, TNAP and CD73, but not AChE, which was followed by those for obese ZF, obese Wistar, lean ZDF, lean ZF and, finally, lean Wistar rats in that ranking order of declining amounts.

The analysis of the potency of structurally different PIGs (for structural details, see Supplementary Materials, Supplementary Figure S5) in displacing GPI-APs (here CD73) from serum GPLD1 (here from obese ZDF rats) revealed that PIG41 was the most efficient, followed by PIG37, PIG45, PIG7 and, lastly, PIG1, in that ranking order of decreasing potency (Figure 8d). The EC_50_ of the PIGs for the half-maximal displacement of full-length GPI-APs from the serum GPLD1 of obese ZDF (Figure 8e) and lean ZF rats (Figure 8f) were the lowest for PIG41 and then increased with PIG37, PIG45, PIG7 and, lastly, PIG1.

The findings that full-length GPI-APs become displaced from serum GPLD1 and presumably other GPI-binding proteins by PIGs raised the possibility of their transfer to acceptor cells upon incubation with serum and PIGs. To test for this, the numbers of GPI-APs expressed at the PMs that had been prepared from the intensively washed acceptor ELCs were measured by chip-based SAW sensing with α-toxin and anti-CD55, CD59, TNAP, AChE and CD73 antibodies.

The PMs from GPI-deficient ELCs incubated with serum from obese ZDF rats (Figure 9a, turquoise curve) compared to its absence (Figure 9a, olive green curve) expressed considerably elevated amounts of total and individual GPI-APs, as reflected in the corresponding successive α-toxin- and anti-CD55, CD59, TNAP and CD73 antibody-induced phase shift increases, respectively. These were further upregulated by the presence of Pha during serum preparation (Figure 9a, dark green curve), PIG41 during serum injection (Figure 9a, black curve) and Pha and PIG41 in combination (Figure 9a, blue curve) in that ranking order of increasing efficacy. This demonstrates the transfer of CD55, CD59, TNAP and CD73 but not AChE from the serum proteins to the ELCs. The transfer was most efficient during the (i) inhibition of serum GPLD1 and concomitant stabilization of the interaction between serum GPI-binding proteins and GPI-APs by Ca^2+^-removal (i.e., Pha) during serum preparation, (ii) its destabilization by Ca^2+^ (i.e., absence of Pha) during serum injection and (iii) displacement of the GPI-APs from the serum GPI-binding proteins by PIG41 during serum injection. Phase shift increases by captured PMs, per se, did not vary significantly under either condition (Figure 9a; 0–300 s). This is compatible with only subtle mass loading onto the chip due to the transfer of GPI-APs and excludes unspecific binding of serum proteins to the PMs. Unspecific binding of serum proteins to the chip channels was assessed by the injection of serum into the chips lacking captured PMs and accounted for only 14 to 17% of the α-toxin- and antibody-induced phase shifts (Figure 9a, brown curve). Nevertheless, the accompanying minor successive phase shift increases confirmed the apparent interaction of serum GPI-binding proteins with GPI-APs, in general, and CD55, CD59, TNAP and CD73 but not AChE, in particular (see Figure 8a,b).

The specificity of the transfer of GPI-APs was corroborated by drastic decreases in α-toxin- and anti-GPI-AP antibody-induced phase shifts upon injection of α-toxin Sepharose beads together with the serum (Figure 9a, yellow curve). Presumably, the beads specifically bound to the GPI glycan core via α-toxin interfered with the insertion of the GPI anchor into the PMs. Pretreatment of the serum with proteinase K (Figure 9a, grey curve) and bacterial PI-PLC (Figure 9a, red curve) led to similar phase shift decreases. The latter condition was used as a background control for the following experiments (Figure 9b,e; blue and black curves).

Importantly, the injection of PIG41 led to reductions of the phase shift increases for each incubation condition (Figure 9a, 1950–2150 s), which thereby compensated the corresponding α-toxin-induced increases (300–500 s). Furthermore, the final injection of TX-100 caused the complete loss of the remaining phase shift increases for each incubation condition (Figure 9a, 2150–2300 s), compatible with the transfer of full-length GPI-APs to the PMs of the GPI-deficient ELCs.

The transfer of GPI-APs from serum to ELCs was strictly dependent on its volume (Figure 9b) and type, i.e., metabolic state of the rats (Figure 9c). The calculation of the volumes effective in stimulating PIG-dependent transfer of GPI-APs by 25% (EV_25_) revealed the following ranking of increasing EV_25_ and, thus, declining potency: Obese ZDF > ZF > Wistar > lean ZDF > ZF > Wistar rats (Figure 9d). Furthermore, stimulation of transfer by the combination of serum and PIGs compared to serum alone was strictly dependent on the structure of the PIGs (Figure 9e). The calculation of their concentrations effective in stimulating serum-dependent GPI-AP transfer by 15% (EC_15_) demonstrated the following ranking order of increasing EC_15_ and, thus, declining potency: PIG41 > 37 > 45 > 7 > 1 (Figure 9f). Taken together, these results strongly argue for the transfer of full-length GPI-APs from rat serum GPI-binding proteins, preferably from those of metabolically dysregulated obese ZDF rats, to GPI-deficient ELCs upon their PIG-induced dissociation.

**Figure 10:**
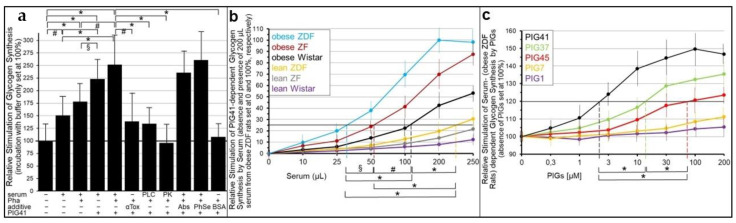
Stimulation of glycogen synthesis in ELCs by PIGs and serum GPI-binding proteins of metabolically dysregulated rats. (**a**–**c**) GPI-deficient EL acceptor cells were incubated (1 week, 37 °C, 5 mM glucose, 1 mM Ca^2+^) in the bottom wells of transwell co-cultures with 200 μL buffer (**a**,**b**) or serum ((**a**,**c**), 200 µL; b, increasing volumes), prepared from obese ZDF rats (**a**,**c**) or rats of different metabolic states (**b**) and then diluted 10-fold with PBS lacking ((**a**), as indicated) or containing 2 mM Pha (**a**–**c**), which were left untreated ((**a**), as indicated; **b**,**c**) or incubated with proteinase K (PK), bacterial PI-PLC (PLC), α-toxin (α-toxin), anti-CD55, CD59, TNAP and CD73 antibodies (Abs), each coupled to Sepharose beads, phenyl Sepharose beads (PhSe) or 200 μL of BSA (4 mg/mL) (**a**), in the absence ((**a**), as indicated) or presence of PIG41 ((**a**,**b**), 30 μM; c, increasing concentrations) or different PIGs (**c**). Thereafter, the ELCs were washed intensely by rinsing the bottom wells three times with 2 mL each of 20 mM Tris/HCl, 1.5 M NaCl and then two times with 2 mL each of PBS and subsequently assayed for glycogen synthesis (15 min, 37 °C, presence of BSA, 0.1 mM [U-^14^C]glucose) as described in Section 4. The experiment was repeated five to seven times (distinct incubations) with determination of glycogen synthesis in triplicate or quadruplicate. (**a**) The relative stimulation of glycogen synthesis by serum and PIG41 in the presence of Pha or other additives is given with incubation with buffer only set at 100%. (**b**,**c**) The relative stimulation of PIG41- (**b**) or serum (from obese ZDF rats)- (**c**) dependent glycogen synthesis by serum from metabolically different rats (**b**) or by different PIGs (**c**), respectively, is shown with the absence and presence of serum set at 0 and 100% (**b**) or the absence of PIGs set at 100% (**c**). Significant differences are indicated between the relative stimulations of glycogen synthesis under the various incubation conditions (**a**) or between the serum volumes effective in 25% (EV_25_, (**b**)) or PIG concentrations effective in 20% (EC_20_, (**c**)) stimulation of glycogen synthesis (horizontal, black lines), as indicated by vertical, hatched, and colored lines (means ± SD; * *p* ≤ 0.01, # *p* ≤ 0.02, ^§^ *p* ≤ 0.05).

These data raised the possibility that glycogen synthesis is stimulated in ELCs upon exposure to serum GPI-binding proteins with bound full-length GPI-APs under conditions that induce their dissociation. The GPI-deficient ELCs incubated with serum from obese ZDF rats (in the presence of Ca^2+^) caused significant stimulation of glycogen synthesis, which was further increased by the presence of Pha during the preparation of the serum, presence of PIG41 and, most potently, by these two conditions in combination (Figure 10a). The elevated glycogen synthesis was reduced by the presence of α-toxin Sepharose beads during the incubation of the serum with the cells, or pretreatment of the serum with bacterial PI-PLC or proteinase K in that ranking order of increasing efficacy. In contrast, anti-CD55, CD59, TNAP, CD73 and AChE antibody Sepharose beads or phenyl Sepharose beads present during the incubation of the serum with the cells (in the presence of Ca^2+^) did not compromise the stimulation of glycogen synthesis by PIG41 combined with serum, which was prepared in the presence of Pha. Finally, BSA failed to substitute for serum in upregulating glycogen synthesis in ELCs in the presence of PIG41 (Figure 10a). This is explained best with the stimulation of glycogen synthesis in GPI-deficient ELCs in the course of the transfer of full-length GPI-APs to their PMs from serum GPI-binding proteins. However, transferred CD55, CD59, TNAP, CD73 and AChE apparently have no effect on glycogen synthesis. Importantly, glycogen synthesis stimulation was fostered most efficiently by the inhibition of serum GPLD1 in parallel to the stabilization of the interaction between serum GPI-binding proteins and GPI-APs by the chelating of Ca^2+^ during the serum preparation in combination with the efficient displacement of the GPI-APs from the serum GPI-binding proteins by PIG41 during serum injection in the presence of Ca^2+^ (Figure 10a).

The stimulation of glycogen synthesis by PIG41 and serum GPI-binding proteins was strictly dependent on the type of serum and its volume (Figure 10b). The calculation of the volumes effective in stimulating PIG-dependent glycogen synthesis by 25% (EV_25_) revealed the following ranking order of increasing EV_25_ and, thus, declining potency of the sera: obese ZDF, obese ZF, obese Wistar, lean ZDF, lean ZF and lean Wistar rats (Figure 10b).

Furthermore, the stimulation of the serum-dependent glycogen synthesis by PIGs was strictly dependent on their structure (Figure 10c). The calculation of the concentrations of PIGs effective in stimulating serum-dependent glycogen synthesis by 20% (EV_20_) demonstrated the following ranking order of increasing EC_20_ and, thus, declining potency: PIG41, -37, -45, -7 and -1 (Figure 10c). Taken together, these data strongly argue for the stimulation of glycogen synthesis in GPI-deficient ELCs upon transfer of full-length GPI-APs from rat serum GPI-binding proteins, preferably from those of metabolically dysregulated rats, upon their PIG-induced displacement.

## 3. Discussion

The major aim of this study was to corroborate a causal relationship between the transfer of full-length GPI-APs to and the induction of anabolic effects in acceptor cells and, in addition, to identify intrinsic and/or extrinsic modulators of GPI-AP transfer, which may hint to its (patho)physiological relevance.

### 3.1. Residence at PMs of Transferred GPI-APs as a Prerequisite for the Induction of Anabolic Effects

First, the kinetics of residence at and disappearance from the PMs of acceptor cells of transferred GPI-APs were compared with the time course of the stimulation of glycogen synthesis (Figure 1 and Figure 2). Transwell co-cultures of human donor adipocytes with GPI-deficient EL acceptor cells under normal conditions (Figure 1) or blockade of internalization of the transferred GPI-APs by chemical inhibitors (Figure 2a–c) or siRNAs (Figure 2d–f) revealed positive correlations between the amount of GPI-APs at PMs of the acceptor cells and the rate of their glycogen synthesis. This strongly argued for a mechanistic link of transfer of GPI-APs to and induction of anabolic effects in acceptor cells, at least under conditions of a low or missing expression of endogenous GPI-APs. The kinetics of the internalization of the transferred GPI-APs seem to be comparable to that of endogenously expressed counterparts in wild-type cells [43,47,65,66]. Thus, internalization of GPI-APs at acceptor cells may lead to underestimation of the rate of GPI-AP transfer and even mask it in case of the high “background” of endogenously expressed GPI-APs in wild-type cells. The use of GPI-deficient acceptor cells and SAW sensing, with its exquisite sensitivity, helped to overcome this issue. Alternative pulse-chase experiments with wild-type acceptor cells and radiolabeled transferred GPI-APs are more complex regarding the design and difficult to interpret due to the unknown pool sizes of the endogenous GPI-APs.

Interestingly, the transfer of GPI-APs to GPI-deficient ELCs significantly increases the glucose sensitivity of the glycogen synthesis machinery and thereby enables the considerable accumulation of glycogen at a blood glucose concentration at the basal state (5 mM) in the absence of typical stimuli of glucose metabolism, such as insulin or antidiabetic SUs. In fact, ELCs do not respond at all to insulin, glimepiride or glibenclamide (see Supplementary Materials, Supplementary Figure S4c,d), most likely due to the fact of the missing expression of the insulin receptor and lipid rafts, respectively, as has already been demonstrated (see [39] and below) and/or the insulin-/SU-dependent GPI-PLC.

### 3.2. “Indirect” Transfer of GPI-APs and Its Control by Insulin and SUs

Insulin and the antidiabetic SUs, glimepiride and glibenclamide, were found to interfere with the transfer of GPI-APs from human donor adipocytes to GPI-deficient EL acceptor cells in a concentration-dependent fashion at physiological and pharmacological concentrations, respectively (Figure 3), and concomitantly with transfer-induced glycogen synthesis (Figure 4). Both insulin and SU inhibition of GPI-AP transfer are most likely due to the induction of lipolytic cleavage of full-length GPI-APs at PMs of the donor adipocytes by insulin-/SU-dependent GPI-PLC. Its activation has previously been demonstrated to be more pronounced with insulin than glimepiride and, lastly, glibenclamide [67,68], which corresponds well to their ranking of the inhibition of GPI-AP transfer and transfer-induced glycogen synthesis.

Unexpectedly, both insulin and SU inhibition were found to be abrogated by serum added to the transwell co-culture (Figure 5, Figure 6 and Figure 7). Previously, the inhibition of GPI-AP transfer and transfer-induced glycogen synthesis by serum, per se, has been reported [30]. Thus, the observation of the considerable transfer (Figure 5 and Figure 6) and transfer-induced glycogen synthesis (Figure 7) in the presence of insulin or SU together with serum seems to be curious, since each of these factors causes inhibition if assayed alone. Serum from obese ZDF rats, which display the most pronounced dysregulation of their metabolic state (see Supplementary Materials, Supplementary Table S2), was the most effective compared to sera from metabolically less dysfunctional rats.

Pretreatment of the serum demonstrated that the full-length GPI-APs that are transferred to (Figure 7) and induced glycogen synthesis (Figure 8) in the acceptor cells in the simultaneous presence of serum and insulin or SUs do originate from serum proteins loaded with full-length GPI-APs, so-called serum GPI-binding proteins, rather than from the donor adipocytes. These findings prompted to differentiate between an “indirect” mode of transfer, with GPI-APs originating from serum GPI-binding proteins with loaded full-length GPI-APs, as assayed by transwell co-culturing in the presence of serum from metabolically deranged rats, and a “direct” mode, with GPI-APs derived from donor cells, as assayed without serum or in the presence of serum from normal rats. Thus, the “indirect” transfer critically depends on the metabolic state of the tissue/organism rather than on the release of full-length GPI-APs from the PMs of donor cells and tissues. So far, serum GPLD1 has been identified as the only GPI-binding protein in serum (Figure 8a) and found to be most abundant and/or pronouncedly loaded with GPI-APs in serum from metabolically dysregulated rats (Figure 8b,c). Recent experimental evidence has suggested that, in addition to GPLD1, certain Ca^2+^-dependent and -independent serum proteins operate as binding entities for full-length, as well as lipolytically cleaved, GPI-APs through the recognition of their highly conserved GPI glycan core [34]. Conversely, the inhibitory potency of serum alone on the transfer of GPI-APs, caused by their Ca^2+^-dependent interaction with the GPI-binding proteins and the resulting prevention from insertion into the acceptor cell PMs, is not significantly affected by the metabolic state (Figure 5). Therefore it is tempting to speculate that only a minor portion of the binding sites of the serum GPI-binding proteins is occupied by (full-length) GPI-APs, provided the levels of insulin or SUs are rather low and concomitantly the rate of release of full-length GPI-APs from PMs in that organism is not very high, i.e., in the case of a normal or only moderately deranged metabolic state. In conclusion, rat serum GPI-binding proteins seem to operate as both donors and acceptors for full-length GPI-APs that are destined for or prevented from transfer, respectively, depending on the metabolic state.

This raised the question concerning the mechanism of integration of the signals elicited by insulin, SUs and the metabolic state for the differential operation of serum GPI-binding proteins as donors and acceptors of full-length GPI-APs and, in consequence, for the (pathophysiological) control of the “indirect” mode of GPI-AP transfer. A crucial finding was the concentration-dependent displacement of serum full-length GPI-APs from GPLD1 by synthetic PIGs, which structurally resemble the glycan core of the GPI anchor (Figure 8d–f). PIG41 displaying the highest structural similarity (see Supplementary Materials, Supplementary Figures S3,S5) was most potent. This led to speculation that GPI-APs lipolytically released from PMs of donor cells upon challenge with insulin or SUs, so-called PIG-proteins, act as physiological competitors for the displacement of full-length GPI-APs from serum GPI-binding proteins, such as GPLD1, thereby initiating their transfer to and anabolic effects in acceptor cells.

This hypothesis was tested by incubation of GPI-deficient ELCs with serum in the presence of PIGs rather than donor cells. In fact, PIG41 and, with reduced potency, PIGs of lower structural similarity to the GPI glycan core managed to elicit GPI-AP transfer (Figure 9) and glycogen synthesis upregulation (Figure 10) in a concentration-dependent fashion. In agreement with serum proteins as a source for full-length GPI-APs transferred upon their displacement, adsorption to α-toxin Sepharose beads and lipolytic or proteolytic digestion of serum (Figure 9a and Figure 10a) eliminated both transfer and glycogen synthesis upregulation, and both were found to be strongly correlated to the serum volume and the metabolic state of the rat donors (Figure 9b–d and Figure 10b), with serum from obese ZDF rats, apparently loaded with the highest amounts of GPI-APs (Figure 8b,c), being most potent. The apparent correlation between PIG-induced GPI-AP transfer and glycogen synthesis further argued for their mechanistic coupling. The components that are specifically involved in this coupling, in particular the GPI-APs transferred and their actions downstream to the glycogen synthesis machinery without involvement of their internalization, remain to be elucidated in future studies, which may continue relying on transwell co-cultures and SAW sensing, in part.

Previous studies have shown that in the absence of serum the release of full-length GPI-APs from the PMs of donor cells in response to endogenous or exogenous stimuli is rate-limiting for “direct” transfer rather than their insertion into the PMs of acceptor cells, which proceeds with fast kinetics [48,69]. Therefore, it is reasonable to assume that also in the presence of serum the observed considerable time requirement for both insulin- and glimepiride-stimulated “indirect” transfer of GPI-APs, as measured in transwell co-cultures (Figure 5e,f), does not rely on the insertion step but rather is caused by the process of their dissociation from the serum GPI-binding proteins upon stimulation of the donor cells by insulin and glimepiride. However, the activation of the insulin-/SU-dependent GPI-PLC and the resulting lipolytic conversion of GPI-APs to PIG-proteins, as the competitors of full-length GPI-APs for binding to the serum GPI-binding proteins, are known to represent rapid processes [22,23,70]. Consequently, de novo synthesis and transport along the secretory pathway of full-length GPI-APs, as precursors for the PIG-proteins, in insulin-responsive donor cells may represent the rate-limiting step for the insulin-/SU-stimulated “indirect” transfer of GPI-APs from serum GPI-binding proteins to acceptor cells.

### 3.3. Contribution of the “Indirect” Intercellular Transfer of GPI-APs to Insulin and SU Action

Both insulin and antidiabetic SUs lower blood glucose by the stimulation of glucose uptake and incorporation into lipids and glycogen in adipose, muscle and liver tissues, albeit through engagement of completely different molecular mechanisms. Insulin activates canonical signaling from the insulin receptor via insulin receptor substrate and phosphatidylinositol-3 kinase (PI-3K) downstream to intracellular Glut4 vesicles, which ultimately fuse with PMs, as well as to key enzymes of glycogen and lipid synthesis, which became activated (for a review, see [71,72]). SUs bind to sulfonylurea receptors at PMs of pancreatic ß-cells, thereby inducing their depolarization and accompanying Ca^2+^-dependent exocytosis of insulin-containing granules (for a review, see [73,74]). Importantly, SUs of the 1st, 2nd and 3rd generations, such as tolbutamide, glibenclamide and glimepiride, respectively, exhibit considerable differences in structure (see Supplementary Materials, Supplementary Figure S2) and pharmacological activity. Tolbutamide lowers blood glucose by stimulation of insulin secretion from pancreatic ß-cells, exclusively [74,75,76]. In contrast, glibenclamide and glimepiride elicit blood glucose decrease by induction of insulin release to a major degree [77,78], as well as to a minor degree by insulin-independent stimulation of transport and nonoxidative metabolism of glucose in adipose [57,68,79,80] and muscle [80,81,82] cells in vitro, and in animals [69,78] and patients [83,84,85,86,87]. Interestingly, with regard to the insulin-independent insulin-mimetic activity at peripheral target cells, glimepiride turned out to be significantly more potent than glibenclamide both in vitro [57,67,68,79,81,88] and in vivo [46,89,90,91,92], whereas glibenclamide is more efficient in releasing insulin (for a review, see [67,84,93,94]).

The molecular mechanism underlying the initiation of this insulin-mimetic so-called extrapancreatic activity of glimepiride has been attributed to engagement of the insulin-/SU-dependent GPI-PLC at PMs of cells at peripheral tissues [22,23,28] and the resulting generation of cleavage fragments derived from free GPI lipids or GPI anchors of GPI-APs with structural similarity to PIGs or PIG-proteins, respectively. Those molecules have been postulated to act as intracellular soluble mediators of metabolic insulin-mimetic action (for a review, see [25,26,51,95,96,97,98]). In fact, the chemically synthesized PIGs used in this study [99,100] or other structurally similar ones [101,102,103] and PIG-proteins, which were prepared from human acetylcholinesterase [104], trypanosomal VSG [105] and yeast Gce1p [106,107], have been demonstrated to stimulate glucose transport and lipid and glycogen synthesis upon incubation of adipocytes, myocytes and hepatocytes in medium containing serum (cultured cells) or (minute amounts of) blood (left in course of preparation of primary cells).

As shown in this study, both insulin and glimepiride stimulate glycogen synthesis in human ELCs but only in transwell co-culture upon simultaneous incubation with adipocytes and serum. ELCs lack both the insulin receptor [39] and lipid rafts (Müller and Müller, unpublished data), which are known to mediate activation of the insulin-/SU-dependent GPI-PLC by glimepiride. Its activation by insulin or glimepiride in insulin target cells has been amply documented, as reflected in the release of GPI-APs, among them CD73, Gce1, lipoprotein lipase and alkaline phosphatase, from the surface of isolated and cultured muscle, liver and adipose cells [22,23,28]. Importantly, its activation by glimepiride does not involve canonical (wortmannin-sensitive) insulin signaling (see Supplementary Materials, Supplementary Figure S4b) but seems to rely on the engagement of a nonreceptor tyrosine kinase anchored at the inner leaflet of PMs, within so-called lipid rafts. Hydrophobic glimepiride molecules have been demonstrated to spontaneously intercalate into this nano- or microdomains [108,109,110]. They are characterized by resistance towards detergent solubilization and low buoyant density due to the presence of high concentrations of cholesterol, glycolipids and GPI-APs equipped with long-chain saturated fatty acids [8] (for a review, see [9,10,11,12]). Glimepiride intercalation leads to the redistribution of the lipid raft constituents including the GPI-PLC and its PI-3K-insensitive activation.

Importantly, the stimulation of lipid synthesis in human adipocytes was completely abrogated by the downregulation of the insulin-/SU-dependent GPI-PLC (Supplementary Materials, Supplementary Figure S4b) using a synthetic inositol derivative, GPI2350, which inhibits bacterial, trypanosomal and serum (G)PI-PLC/D with high potency and selectivity and reduces the insulin-/SU-inducible lipolytic release of GPI-APs from the surface of intact rat adipocytes [52,92]. In contrast, the inhibition of the insulin-/SU-dependent GPI-PLC failed to interfere with the insulin stimulation of lipid synthesis in human adipocytes (Supplementary Materials, Supplementary Figure S4a). Thus, it is tempting to speculate that activation of the insulin-/SU-dependent GPI-PLC is a prerequisite for the induction of anabolic effects in insulin nontarget cells, such as ELCs, or in cells lacking insulin receptor or with defective insulin signaling, rather than in insulin target cells. Thus, insulin and glimepiride share activation of the insulin-/SU-dependent GPI-PLC in adipocytes, resulting in the release of PIG-proteins from full-length GPI-APs of adipocyte PMs. These PIG-proteins apparently operate as physiological competitors for the displacement of (nonadipocyte) full-length GPI-APs from serum GPI-binding proteins rather than as intracellular mediators of insulin-like action as originally thought (see for this view [25,26,96,97]). The displaced full-length GPI-APs subsequently transferred to PMs of acceptor cells, rather than the generated PIG-proteins themselves induce the reported anabolic effects. Thus, glimepiride seems to exert its insulin-mimetic effects via the “indirect” mode of GPI-AP transfer. Importantly, in both in vitro [67,68,79,80,81,94] and clinical studies [85,86,87] the insulin-mimetic action on peripheral cells and tissues has been shown to be more pronounced for glimepiride compared to glibenclamide and tolbutamide. The same ranking order of declining potency for SUs of the 3rd, 2nd and 1st generations was found in the present study for the stimulation of GPI-AP transfer (Figure 6a,b) and glycogen synthesis (Figure 7c,d) in the presence of serum. These correlations represent important hints to the relevance of the “indirect” mode of transfer of GPI-APs from serum GPI-binding proteins to peripheral cells for the insulin-independent (extrapancreatic) blood glucose-lowering activity of antidiabetic SUs which, however, must be further delineated in future animal studies.

At variance with glimepiride, insulin exerts its metabolic effects in insulin target cells predominantly via engagement of canonical insulin signaling. This conclusion was exemplified best by the lacking effect of GPI2350 on insulin stimulation of lipid synthesis in 3T3 adipocytes [52], which excludes a major role of the insulin-/SU-dependent GPI-PLC in metabolic insulin signaling and action in insulin target cells (see Supplementary Materials, Supplementary Figure S4a). The minor portion of the anabolic effects of insulin mediated by the “indirect” mode of GPI-AP transfer is most likely over-run by the considerably more potent canonical signaling to and activation of the anabolic effector systems.

### 3.4. The Interplay between the “Indirect” and the “Direct” Modes of GPI-AP Transfer

The differentiation between the “indirect” mode of GPI-AP transfer in the presence of serum GPI-binding proteins and insulin or antidiabetic SUs, as described in the present study, and the “direct” mode in their absence, as reported previously [30], raises the possibility that the two modes are linked to distinct physiological roles. The requirement for the absence of serum GPI-binding proteins for the “direct” mode suggests that this GPI-AP transfer occurs between cells of the same tissue depot and over short distance (i.e., across interstitial spaces), exclusively and solely depends on the efficacy of the release of full-length GPI-APs from donor cells and insertion into PMs of acceptor cells in the immediate neighborhood. For both steps, the size and metabolic state of the donor and acceptor cells has previously been found to be critical, with those of large and small sizes or from metabolically deranged and normal rats favoring the release and insertion, respectively [30,47]. In the case of adipose and liver tissue depots, GPI-AP transfer may result in the upregulation of basal lipid and glycogen synthesis, respectively, in “empty” acceptor cells, which take over the burden of lipid and glycogen storage from the neighboring donor cells with completely filled lipid and glycogen stores, respectively. In fact, the heterogeneity of the size and metabolic state of cells within the same adipose or liver tissue depots has been amply documented [111,112]. Thus, the “direct” mode of GPI-AP transfer may be regarded as a mechanism to compensate for the unequal distribution of glycogen and lipid synthesizing capabilities, which could be associated with unequal expression of “relevant” GPI-APs, between different liver or adipose cells within the same tissue depots. Furthermore, upregulation of lipid and glycogen synthesis in insulin target tissues, such as adipose and liver, in response to the “direct” transfer of GPI-APs may be interpreted as a mechanism to override peripheral insulin resistance in type II diabetic patients to a certain (limited) degree.

The “Indirect” transfer of full-length GPI-APs from donor to acceptor cells located at distinct tissue depots over long distance along the circulation engages the interaction with and dissociation from serum GPI-binding proteins and, therefore, depends on several parameters: (i) concentration of full-length GPI-APs in blood, which is determined by their release from tissues in response to the metabolic state; (ii) concentration of GPI-binding proteins in blood; (iii) concentration of lipolytically cleaved GPI-APs (i.e., PIG-proteins) in blood, which is determined by the activation state of the insulin-/SU-dependent GPI-PLC; and (iv) efficacy of the insertion of full-length GPI-APs into the acceptor cell PM.

Insulin and/or SUs will block the “direct” mode of GPI-AP transfer in the course of the lipolytic removal of their GPI anchor and in parallel stimulate the “indirect” mode by inducing the dissociation of full-length GPI-APs from serum GPI-binding proteins. Thus, the insulin-, SU- and metabolic state-controlled integration of parameters (i–iv) will ultimately determine the “direct” vs. the “indirect” mode of GPI-AP transfer. In the metabolically dysregulated state, such as type II diabetes and obesity, the “indirect” mode will override the “direct” one due to the (i) upregulation of the release of full-length GPI-APs from PMs of metabolically dysregulated (insulin target) cells and tissues (e.g., adipose and liver); (ii) interaction of those with serum GPI-binding proteins, which are systemically distributed along the circulation; (iii) generation of lipolytically cleaved GPI-APs (PIG-proteins) by insulin-/SU-dependent GPI-PLC in the hyperinsulinemic/hyperglycemic state and/or insulin or glimepiride therapy, which displace full-length GPI-APs from the serum GPI-binding proteins; and (v) final insertion of the full-length GPI-APs into PMs of metabolically dysregulated acceptor cells, including noninsulin responsive or noninsulin target cells.

Thus, it is conceivable that the “indirect” mode of GPI-AP transfer operates in insulin responsive, as well as nonresponsive cells, i.e., in acceptor cells lacking canonical insulin signaling and located far away from the insulin responsive donor cells. It may thereby lead to the stimulation of glycogen and lipid synthesis that is additive to and independent of canonical insulin signaling. In the future, it will be interesting to investigate whether other hormonal and therapeutic signals or extrinsic factors affect the “direct” and “indirect” modes of the intercellular transfer of GPI-APs and the resulting shift of the acceptor cell phenotype. Oxidative challenge may represent a (patho)physiological candidate and this even more so because an important role of glucose as a major antioxidant, in general, and of glycogen stores in the resistance of cells towards oxidative stress, in particular, has been suggested [113].

Moreover, it remains to be clarified (i) whether cellular phenotypes other than the anabolic state become transmitted by intercellular protein transfer, (ii) what cells/tissues are involved, (iii) what mechanisms (“direct” or “indirect”) are engaged and (iv) what GPI-APs are transferred. Regarding the induction of the anabolic phenotype in ELCs and adipocytes, the present data strongly argue that the relevant GPI-APs are different from CD55, CD59, TNAP, CD73 and AChE, which are transferred along with the phenotype, however, apparently without any mechanistic coupling to it. Unfortunately, the repertoire of commercially available antibodies against GPI-APs, suitable for their immune depletion during transwell co-culture or from serum GPI-binding proteins with resulting blockade of transfer in parallel to phenotypic changes in the acceptor cells, is rather limited. Thus, an experimental alternative may represent downregulation of individual GPI-APs by siRNAs in donor cells of transwell co-cultures at large scale if the lack of their expression is compatible with donor cell viability.

## 4. Materials and Methods

### 4.1. Ethical Approval

All experimental procedures regarding the handling of animals (housing of and blood sampling from rats, see [56]) were conducted in accordance with the German Animal Protection Law (paragraph 6) and corresponded to international animal welfare legislation and rules.

### 4.2. Transwell Co-Culture of Human Adipocytes and GPI-Deficient ELCs

Transwell co-culture was used with GPI-deficient ELCs as acceptor cells seeded at the bottom of 12-well tissue culture plates (Falcon Companion TC Plate, No. 353503, Falcon/Corning, Tewksbury, MA, USA, for 1.4–2.3 mL medium) and human adipocytes of lipid-loading stage II or IV [30], differentiated from hADSCs (see below), as donor cells seeded in the 12-well cell culture inserts (Falcon Cell Culture Insert, No. 353103, for 0.4–1.0 mL medium). This enabled the detection of the transfer of full-length GPI-APs between donor and acceptor cells at a distance (from the membrane to the bottom of wells) of 0.9 mm through a porous membrane (pore size 1.0 µm, high pore density 1.6 ± 0.6 × 10^6^ pores/cm^2^, polyethylene terephthalate track-etched, transparent).

The hADSCs were expanded in “hADSCs Growth Medium” (iXCells Inc., San Diego, CA, USA) for 3–4 passages, as described in detail previously [30,114], and then seeded at 5 × 10^3^ cells/cm^2^ in the transwell inserts (12-well plate formate) and thereafter grown to confluence with medium change every 2–3 days until the cells reached 70–80% confluence. For this, a cell culture insert was removed from the package with sterile forceps and then gently placed into the bottom companion well culture plate, taking care to avoid trapping air under the insert by tilting the insert while lowering it onto the well. Upon correct positioning, the inserts with the flanges rested in the notches on the top edge of each well in diagonal arrangement. For seeding, the cells and 1 mL of medium were added to the cell culture insert at the density given above and cultured under routine conditions.

Thereafter, hADSCs were differentiated into human adipocytes in vitro using “hADSC Adipocytes Differentiation Medium” (iXCells Inc.), as reported previously [30], until medium or heavy lipid-loading (stage II or IV, respectively) was reached (i.e., Oil Red-stained lipid droplets accounted for 50% or more than 80% of the cytoplasmic area, respectively). Following washing with Dulbecco’s modified Eagle medium (DMEM, Gibco-BRL, Thermo Fisher Scientific, Waltham, MA, USA) containing 1% sodium pyruvate, 100 U/mL of penicillin and 100 µg/mL of streptomycin, the human adipocytes were used for transfer experiments (lipid-loading stage IV for routine use, stage II as indicated).

Mutant ELCs, incapable of the coupling of nonacetylated glucosamine to phosphatidylinositol during GPI anchor biosynthesis and, thus, completely deficient in expression of GPI-APs [35,39], were seeded at 0.3–1.2 × 10^6^ cell/mL in the bottom wells (12-well formate) in RPMI 1640 medium supplemented with 10% FBS and 1% penicillin/streptomycin (DMEM, Gibco-BRL, Thermo Fisher Scientific, Waltham, MA, USA) and grown to confluence. The ELCs were washed two times with Ca^2+^-free PBS and then two times with serum-free medium for transfer experiments.

For initiation of transfer, the inserts were first moved to one side using a sterile 1 mL pasteur pipet to remove media from above and below the membrane. Subsequently, 2 mL and 1 of fresh medium containing or lacking serum, as indicated, were added to the wells of the bottom companion and insert cell culture plates, respectively. Following incubation under the conditions indicated, the insert wells were removed and then the medium was aspirated from the bottom wells. Thereafter, the ELCs of the bottom wells were rinsed two times with 1 mL each of PBS and then used for the preparation of PMs to determine the amount of GPI-APs for the assay of GPI-AP transfer or for incubation with D-[U-^14^C]glucose (PerkinElmer, Waltham, MA, USA) to assay glycogen synthesis.

### 4.3. Assay of GPI-AP Transfer

PMs were prepared from the ELCs in the bottom wells of transwell co-culture, as described previously [30], and then immobilized at the chip surface by ionic and covalent capture with high efficacy. For ionic capture, PMs (0.2 mg protein per mL of 2 mM Ca^2+^, 100 mM NaCl, 10 mM Hepes/NaOH, pH 7.5) containing positively charged, negatively charged or zwitterionic phospholipids or combinations thereof were injected into the chips with uncoated negatively charged and highly hydrophilic TiO_2_ channels together with immobilization buffer (10 mM sodium acetate, pH 5.5) at a flow rate of 25 μL/min for 4 min at 30 °C, which fostered the formation of salt bridges between the chip surface and the PM phospholipids. After the termination of the flow for 20 min at 30 °C for the stabilization of immobilization, the chips were washed with 10 mM Hepes/NaOH (pH 7.5) and 100 mM NaCl at a flow rate of 150 μL/min for 20 min at 30 °C.

For the subsequent covalent capture via the protein moieties of GPI-APs, as well as the extracellular domains of TMPs, the microfluidic channels of the uncoated TiO_2_ chips were primed with three injections of 250 μL each of immobilization buffer at a flow rate of 50 μL/min. Then, the channel surface was activated by a 250 μL injection of 0.2 M EDC and 0.05 M Sulfo-NHS (Pierce/ThermoFisher Scientific, Waltham, MA, USA; mixed from 2×-stock solutions right before the injection) at a flow rate of 50 μL/min. After a waiting period of 3 min (flow rate 0) and subsequent washing of the channels with two 300 μL portions of PBS containing 2.5 mM EGTA (PBSE) at a flow rate of 180 μL/min, the residual activated groups on the channel surface were capped by injecting 200 μL of 1 M ethanolamine (pH 8.5) at a flow rate of 60 μL/min. Thereafter, the channels were washed two times with 125 μL of PBSE each at a flow rate of 150 μL/min and then two times with 160 μL of 10 mM Hepes/NaOH (pH 7.5) each at the same flow rate.

The amounts of GPI-APs and TMPs were determined by sequential injection of 75 μL of appropriate antibodies (diluted as given in Supplementary Materials) at a flow rate of 15 μL/min according to the order indicated in the figures. Finally, for demonstration of anchorage by GPI at the immobilized PMs of the acceptor ELCs, 75 μL of PI-PLC (*Bacillus cereus*, 5 ng; Merck/Sigma-Aldrich, Darmstadt, Germany) at a flow rate of 15 μL/min were injected, followed by injection of three portions of 220 μL of 0.1% (*w*/*v*) Triton X-100 at a flow rate of 200 μL/min for the demonstration of incorporation into the phospholipid bilayer of the immobilized PMs. Phase shifts are given upon correction for unspecific interactions (no PMs immobilized) and normalization for the varying efficacy in immobilization of the PMs between different chips [33].

### 4.4. Assay of Glycogen Synthesis

After disassembly of the transwell co-culture, the ELCs of the bottom wells were washed two times with KRB containing 0.1% BSA and then incubated (120 min, 37 °C) with 0.5 mL of the same buffer containing D-[U-^14^C]glucose (PerkinElmer, Waltham, MA, USA, 250-360 mCi/mmol) and 0.1 to 15 mM glucose, as indicated, and a constant specific radioactivity (0.1 to 15 μCi per test) in a shaking water bath, as has been previously described [30]. The assay was terminated by placing the bottom plate on ice (20 min) and subsequent three washings of the cells with 1 mL of ice-cold PBS each. Thereafter, the ELCs were detached with Accutase (Merck/Sigma-Aldrich, Darmstadt, Germany) and then homogenized (0 °C, ten up- and down-pipettings with a 0.1-mL pipette) in 0.1 mL of 25 mM Tris/HCl (pH 7.4), 5 mM EDTA, 100 mM NaF and 0.1 mM PMSF. The homogenate was then centrifuged (10,000× *g*, 20 min, 4 °C). Then, 40 µL aliquots of the supernatant were transferred into new 2.5 mL tubes, supplemented with 20 µL of 5 mg/mL “carrier” glycogen and 1 mL of 30% KOH, and then heated (45 min, 100 °C) and subsequently adjusted to 70% ethanol by the addition of 1.5 mL of 100% ethanol. After incubation (4 h, −20 °C), the samples were centrifuged (2000× *g*, 15 min, 4 °C). The precipitated glycogen pellets were washed four times with 70% ethanol and then dissolved in 200 µL of distilled water. Three 60 µL portions were spotted on 2 cm^2^ filter papers, which were dried before liquid scintillation counting. The amount of glucose incorporated into glycogen was calculated for each aliquot of the homogenate after the subtraction of the radioactivity measured for a “mock” incubation of cells with D-[U-^14^C]glucose together with KOH and subsequent identical processing.

### 4.5. siRNA Transfection of Human Adipocytes

Human adipocytes were transfected with siRNAs targeting human CDC42, Rac1 or RhoA gene with lipofectamine RNAiMAX according to the manufacturer’s instructions (ThermoFisher Scientific, Waltham, MA, USA). For control of the silencing efficacy, total RNA was extracted for qPCR determination at 48 h after transfection. This protocol was based on the SYBR Green detection system. Primers were used at 10 pM each. The mix included 10 μL of SYBR Green qPCR Mix, 0.4 μL of each primer, 8.2 μL of sterile PCR grade water. Next, 1 μL of template cDNA was added in a final volume of 20 μL. The samples were amplified as follows: an initial denaturation step at 95 °C for 2 min, followed by 40 cycles at 95 °C for 15 s (denaturation) and 60 °C for 1 min (annealing and elongation). After amplification, melting curve analyses were performed to evaluate the silencing efficacies in comparison to scrambled siRNA, which were considered to be 0%.

### 4.6. Immobilization of Anti-GPLD1 Antibody at the Chip Surface

For the generation of chips that capture GPLD1, a 200 µL portion of protein A (50 mg/mL in PBS, 0.1 mM EDTA, 10% glycerol) diluted 10-fold in immobilization buffer (10 mM sodium acetate, pH 5.5) was injected at a flow rate of 40 µL/min into the channels of activated (0.2 M EDC and 0.05 M Sulfo-NHS, mixed from 2×-stock solutions right before injection) long-chain 3D carboxymethyl (CM) dextran chips (SAW Instruments Inc., Bonn, Germany) in a SamX instrument (SAW Instruments Inc., Bonn, Germany). The residual activated groups on the chip surface were capped by injecting 100 µL of 1 M ethanolamine (pH 8.5) at a flow rate of 60 µL/min. Thereafter, 50 µL of monoclonal anti-GPLD1 antibody (diluted 1:750 with running buffer) or as a “blank” control IgG (same concentration) were injected at a flow rate of 15 µL/min. After washing with 200 µL of PBST at a flow rate of 120 µL/min, 100 µL of serum, diluted 20-fold with PBST, was injected at a flow rate of 30 µL/min. Measurement of the phase shift was performed at 22 °C. The start and termination points of the sample injections or washing cycles are indicated with green and black arrows, respectively, in the figures. The chips were regenerated by successive injections of 60 µL of 10 mM glycine (pH 3.5) and 30 µL of 4 M urea with waiting for 5 min after each injection and the final injection of 300 µL of regeneration buffer (PBS, pH 7.4, 1 M NaCl, 0.03% Tween and 0.5% glycerol) and 300 µL of PBST. Chips with immobilized protein A were used to up to 24 times without significant loss of capacity of capturing of GPLD1.

### 4.7. Statistical Analysis

All numerical data are presented as the means ± SD. The statistical significance was calculated using GraphPad Prism6 software (version 6.0.2, GraphPad Software, New York City, NY, USA) on the basis of ordinary one-way/two-way ANOVA, tested by Sidak’s multiple comparison tests. *p* ≤ 0.05 was considered to be significant.

### 4.8. Miscellaneous

The preparation of PMs [92], protein determination [57] and SAW sensing with long-chain 3D CM-dextran sam^®^5 chips using a SamX instrument (SAW/Nanotemper, Bonn/Munich, Germany) and evaluation [56,64] were performed as previously described in detail.

## 5. Conclusions

The main findings of the present study are as follows: (i) the transfer of GPI-APs from human adipocytes to blood cells stimulates basal glycogen synthesis; (ii) insulin and antidiabetic SU drugs inhibit the transfer and stimulation of glycogen synthesis; (iii) serum GPI-binding proteins counteract the insulin and SU inhibition of transfer and glycogen synthesis stimulation; (iv) GPI-AP transfer proceeds via two (i.e. “direct” and “indirect”) modes that differ regarding the absence or presence of serum GPI-binding proteins, respectively, and the origin of the transferred full-length GPI-APs (donor cells or serum GPI-binding proteins, respectively).

It may be insightful to compare the “indirect” mode of transfer of GPI-APs, which presumably reflects the physiology of mammalian organisms, with other modes of the intercellular transfer of membrane proteins, in general, and GPI-APs, in particular: (i) the “direct” mode of GPI-AP transfer lacks control by exogenous cues, such as serum proteins, hormones, and drugs, and only operates between cells of the same tissue depot and over short distance; (ii) for the transfer of membrane proteins via extracellular vesicles between the same or different cell types/tissues over a short or long distance, a control by exogenous signals has not been described so far; (iii) the same holds true for the short-distance delivery of the complete set of membrane systems including their constituting membrane proteins, among them PMs, endoplasmic reticulum, mitochondria, etc., from somatic mother to daughter cells along cell division or from gametes to zygotes along cell fusion. Thus, the “indirect” mode of the intercellular transfer of full-length GPI-APs may be of special biological relevance within the repertoire of nongenetic inheritance of biological matter since it is under control of environmental factors.

Meanwhile, many studies have addressed the role of the protein vs. the GPI moieties of GPI-APs in human health and disease (e.g., [115]; for a review, see [9,116]). Furthermore, the interaction with and displacement by PIGs from human serum proteins, among them GPLD1, of full-length human GPI-APs, among them CD55, TNAP and AChE, have been demonstrated (Müller and Müller, manuscript in preparation). Those findings together with the present ones obtained with human cells and serum from a rat model for a human disorder may foster future experimentation as to whether the (“indirect” and/or “direct”) intercellular transfer of GPI-APs has (a) (patho)physiological role(s) in humans.

## Data Availability

The datasets generated and analyzed during the current study are available from the corresponding author (G.A.M.; guenter.mueller@helmholtz-muenchen.de) upon reasonable request and will be provided as the original SAW data files together with the appropriate SAW Inc., software for data visualization and processing (correction and normalization), if required, under consideration of the relevant conditions for licensing of FitMaster^®^, SensMaster^®^ and SequenceMaster^®^.

## Supplementary Materials

The following supporting information can be downloaded at: https://www.mdpi.com/article/10.3390/ijms24054825/s1. Reference [117] is cited in the supplementary materials.
