# Supplementary material for: Transfer of Proteins from Cultured Human Adipose to Blood Cells and Induction of Anabolic Phenotype Are Controlled by Serum, Insulin and Sulfonylurea Drugs
Source: Int J Mol Sci. 2023 Mar 2;24(5):4825. doi: 10.3390/ijms24054825 (PMC10003403; doi:10.3390/ijms24054825)
Supplement: Supplementary file 1 [file ijms-24-04825-s001.zip › ijms-2098006-supplementary.pdf]

Supplementary Materials for

Transfer of Proteins from Cultured Human Adipose to Blood Cells and Induction of Anabolic Phenotype Are Controlled by Serum, Insulin and Sulfonylurea Drugs

By Günter A. Müller and Timo D. Müller

Materials

Human adipose derived stem cells (hADSCs) were delivered by iXCells Biotechnologies (San Diego, USA, Cat. Nr. 10HU-001). RPMI 1640 medium was obtained from GIBCO (ThermoFisher Scientific, Waltham, MA, USA). The siRNAs specifically targeting human Cdc42, Rac1, RhoA, and universal negative control siRNA (scrambled, nonsense) were obtained from RiboBio Inc. (Guangzhou, China). Lipofectamine RNAiMAX was from ThermoFisher Scientific (Waltham, MA, USA). 1-ethyl-3-[3-dimethylaminopropyl]carbodiimide (EDC) and N-hydroxysulfosuccinimide (Sulfo-NHS, premium grade) were bought from Pierce/ThermoFisher Scientific (Rockford, IL, USA). Chlorpromazine (hydrochloride) and accutase were purchased from Merck (Darmstadt, Germany). Filipin III (Streptomyces filipinensis) (Cat. No. F4767), dynasore, BSA (fraction V, defatted), human glucagon (Cat. No. G2044), human FGF-21 (recombinant, expressed in E. coli; Cat. No. SRP4066) and human serum (human male AB plasma, USA origin, sterile-filtered, 40-90 mg protein/mL, 0.3 mg hemoglobin/mL, MDL MFCD00165829, Cat. No. H4522) were delivered by Merck/Sigma-Aldrich (Darmstadt, Germany). Human (recombinant) insulin, glimepiride, glibenclamide, tolbutamide and meglitinide (all synthesized as reported [79]), PIGs (synthesized as reported [99]) and GPI2350 (synthesized as reported [52]) were kind gifts from Sanofi Pharma Germany GmbH (Diabetes group, Frankfurt am Main, Germany). Other materials (highest purity available) were obtained as described previously [30,33,34,48,56,64].

Antibodies against human Rac1 (rabbit polyclonal, IgG, protein A-purified; raised against recombinant full-length protein corresponding to human Rac1; Cat. No. ab155938; 1:1500), human RhoA (mouse monoclonal, IgG1, purified from tissue culture supernatant; raised against recombinant full-length protein corresponding to human RhoA aa1-193; Cat. No. ab54835; 1:1250), CD55 (ab253284, mouse monoclonal, protein G-purified from tissue culture supernatant, IgG2a, prepared against a recombinant fragment corresponding to the extracellular domain of human CD55; Cat. No. ab253284; 1:1000), TNAP (rabbit polyclonal, affinity-purified, IgG isotype, prepared against an unconjugated synthetic peptide corresponding to TNAP; Cat. No. ab954462; 1:750), CD59 (rabbit

monoclonal, protein A-purified, IgG isotype, prepared against a synthetic peptide corresponding to human CD59; Cat. No. ab248625; 1:2500), AChE (goat polyclonal, multi-step purified, IgG isotype, biotinylated, prepared against purified AChE from bovine erythrocytes; ab34533; 1:1500), Band-3 (rabbit monoclonal, protein A-purified, IgG, prepared against a synthetic peptide corresponding to a fragment of rat Band-3; ab108414; 1:2000), Glut4 (ab216661, rabbit polyclonal, protein A-purified, IgG, prepared against a synthetic peptide corresponding to aa 467-509 of mouse Glut4; ab216661; 1:2500), Glut1 (rabbit polyclonal, immunogen affinity-purified, IgG, prepared against a synthetic peptide corresponding to aa 481-492 of human Glut1; ab14683; 1:400), GPLD1 (1:750) (rabbit polyclonal, IgG, immunogen affinity-purified, raised against a recombinant fragment corresponding to human GPLD1 aa24-160; Cat. No. ab210753) was purchased from Abcam Inc. (Berlin, Germany). Antibody against human Cdc42 (rabbit polyclonal, IgG, protein A-purified; raised against recombinant carboxy-terminally truncated human protein corresponding to human RhoA; Cat. No. PA1-092; 1:2000) was delivered by Thermofisher Scientific (Waltham, MA, USA). Antibodies against human CD73 (mouse monoclonal, prepared against CD73 purified from human placenta with no cross-reactivity against mouse/rat CD73; 1:1000; Cat. No. sc-32299) and human GPLD1 (1:1200) (mouse monoclonal, IgG2a, immunogen affinity-purified, raised against a recombinant fragment corresponding to human GPLD1 aa1-300, D-10; sc-365096) were delivered by Santa Cruz Biotechnology Inc. (Dallas, Texas, USA). Antibody against human Cav1 (rabbit polyclonal, purified by protein A and peptide affinity chromatography, IgG, prepared against a synthetic peptide corresponding to residues surrounding Glu20 of human Cav1; Cat. No. #3238; 1:1000) was obtained from Cell Signalling Technology Europe (Leiden, The Netherlands).

12-((7-nitrobenz-2-oxa-1,3-diazol-4-yl)amino)dodecanoic acid (NBD-FA) was purchased from Molecular Probes Inc. (Eugene, OR, USA). Wortmannin, plates for thin layer chromatography (TLC) (Si-60) and tetrahyrofurane (THF) were from Merck (Darmstadt, Germany). D-[U-^{14}C]glucose (250-360 mCi/mmol, 3% ethanol) (Cat.No. NEC042V), liquid scintillation cocktail Ultima Gold™ MV (Cat.No. 6013151) and the liquid scintillation counter TRI-CARB 4810TR 110V were obtained from PerkinElmer (Waltham, MA, USA).

Methods

S1. Assay for Lipid Synthesis in Human Adipocytes

Lipid synthesis with human adipocytes differentiated from hADSCs in the wells of the upper insert plate of Transwell co-culture (bottom companion plate was left without cells) measured as the incorporation of 12-((7-nitrobenz-2-oxa-1,3-diazol-4-yl)amino)dodecanoic acid (NBD-FA) into total fluorescent acylglycerols. For this, the culture medium was removed by suction from the bottom companion tissue culture plates and replaced by 1 mL/well of 25 mM Hepes free acid, 25 mM Hepes

sodium salt (pH 7.2), 80 mM NaCl, 1 mM $MgSO_4$, 2 mM $CaCl_2$, 6 mM KCl, 1 mM glucose (KRH) containing 0.75% (w/v) BSA (KRHLB). After incubation of the transwell co-culture (30 min, 37 °C, mild shaking [stage 11, thermomixer, Eppendorf, Germany]), the assay was initiated by the addition of 250 µL of 0.9 mM NBD-FA (prepared daily from a 100-mM stock solution in ethanol by dilution with KRHLB under mild heating). After incubation (3 h, 37 °C, as above), lipid synthesis was terminated by suction of the medium from the bottom companion plates and washing of the cells three times with 2 mL/well of KRHLB. After removal of the last washing fluid, the adipocytes were dissolved and their acylglycerols were extracted by addition of 1.5 mL/well of tetrahydrofurane (THF) to and rigorous shaking (20 min) of the upper insert plate. Thereafter, 1.4 mL of the extract was transferred into new tubes and centrifuged (15,000x *g*, 5 min). The supernatant was transferred into new tubes, then dried (SpeedVac) and finally suspended in 50 µL THF. 5-µL samples were analyzed by thin layer chromatography (TLC) on silica gel Si-60 plates using 78 mL diethylether, 22 mL petrol ether, 1 mL acetic acid as solvent system. Fluorescent (acylglycerol) products on the dried plates were visualized and quantitatively evaluated by fluorescence imaging (Storm 860 phosphorimager, Molecular Dynamics, Krefeld, Germany) with excitation at 460 nm and emission at 540-560 nm. After subtraction of a background value (derived from an equal-sized region of a lane of the TLC plate which was used for spotting of a lipid-containing sample, but did not contain any lipidic product), the sum of the relative peak areas corresponding to all fluorescently labeled acylglycerols was calculated (arbitrary units) and used as measure for total lipid synthesis.

S2. Solubilization of SUs

A 10-mM stock solution of glimepiride (made daily) was prepared by suspending 9.95 mg of glimepiride in 1.94 mL aqua bidest., followed by addition of 60 µL of 1 M NaOH and warming-up to 60-70 °C. After dilution of the stock solution with 25 mM Hepes/KOH (pH 7.4), solubilized glimepiride was added to the culture medium at the desired final concentration and dilution of 1:200 dilution at least.

Excess glibenclamide (50 mg) was added to 10 mL of 0.48% Tween 80, 8.5% EtOH (pH 7.4) and incubated (24 h, 37 °C) in a shaking water bath. After an equilibration period (12 h, 37 °C), aliquots were filtered using a 0.22-µM Millipore membrane filter. This stock solution was added directly to the culture medium, resulting in the desired final concentration.

10-mM stock solutions of tolbutamide and meglitinide were prepared with 25 mM Hepes/KOH (pH 7.4).

Concentrations of the sulfonylureas in the culture media were analyzed by high-performance liquid chromatography with ultraviolet, evaporative light scattering or charged aerosol detections as described previously [117].

Supplementary Tables

Supplementary Table S1. Quantitative evaluation of the efficacy and specificity of silencing of Cdc42, RhoA and Rac1 proteins in erythroleukemia cells (ELCs). The experiment was performed as described for Supplementary Figure S3. The expression of each membrane protein left after incubation of the wildtype ELCs with individual siRNAs, combined siRNAs and scrambled siRNAs relative to the absence of siRNA (set at 100 arbitrary units) is given (means ± S.D.), with significant differences of the presence of individual, combined or scrambled siRNA vs. buffer indicated (* $p \leq 0.01$). Data for homologous configurations, i.e. silencing of and SAW sensing for the same (individual or total) protein(s) are given in bold.

siRNA	buffer	Cdc42	RhoA	Rac1	Cdc42 + RhoA + Rac1	scrambled
**Cdc42 protein**	100 ± 13	**37 ± 5***	90 ± 15	103 ± 10	**40 ± 7***	96 ± 14
**RhoA protein**	100 ± 16	105 ± 18	**32 ± 3***	87 ± 15	**35 ± 6***	85 ± 12
**Rac1 protein**	100 ± 9	92 ± 8	104 ± 23	**27 ± 6***	**29 ± 4***	83 ± 16
**Glut-1 protein**	100 ± 15	98 ± 23	103 ± 19	95 ± 15	86 ± 11	111 ± 21
**Cav-1 protein**	100 ± 13	89 ± 12	91 ± 10	88 ± 19	90 ± 25	93 ± 16
**CD55 protein**	100 ± 19	94 ± 17	98 ± 21	97 ± 24	82 ± 20	105 ± 26

For interpretation of the data, see Supplementary Materials, Supplementary Figure S3.

Supplementary Table S2. Characteristics of the six rat groups (n = 12-16). Weight, fasting blood glucose and fasting plasma insulin for each rat group (of a given genotype and feeding state) are shown (means + S.D.; * $p \leq 0.01$, § $p \leq 0.02$, # $p \leq 0.05$ vs. lean Wistar). Male Wistar rats (Crl:WI(WU)), male Zucker diabetic fatty rats (ZDF-*Lepr*^{fa}/Crl) and male Zucker fatty rats (Crl:ZUC(Orl)-*Lepr*^{fa}/Zucker) were obtained from Charles River (Sulzfeld, Germany). Rats were housed two *per* cage in an environmentally controlled room with a 12:12-h light-dark circle (light on at 06:00) and *ad libitum* access to food and water. Standard chow (17.7 kJ/g, Ssniff diet R/M-H, V1535 with 18% crude protein, 4.7% sugar, and 3.5% crude fat) and a cafeteria diet (21.6 kJ/g, Ssniff diet EF R, 10 mm with 13.5% crude protein, 42.8% sugar, and 22.5% crude fat) (Sniff, Soest, Germany) were used for induction of the lean and obese phenotype, respectively. All experimental procedures were conducted in accordance with the German Animal Protection Law (paragraph 6) and corresponded to international animal welfare legislation and rules.

Geno-type	Feeding State	Weight (g)	Age (week)	Fasting Blood Glucose [mM]	Fasting Plasma Insulin [µg/L]	Metabolic Phenotype
Wistar	lean	281.5 + 36.7	12	6.21 + 0.35	0.88 + 0.23	Normoglycemic Normoinsulinemic
	obese	489.1 + 67.8*	12	6.83 + 0.55	1.90 + 0.29*	Normoglycemic mildly hyperinsulinemic
ZF	lean	430.4 + 52.7*	38	5.12 + 0.62#	0.98 + 0.32	Normoglycemic Normoinsulinemic
	obese	712.5 + 85.2*	38	6.03 + 0.69	3.64 + 0.50*	Normoglycemic Hyperinsulinemic
ZDF	Lean	326.1 + 49.4	15	5.84 + 0.59	1.44 + 0.38#	Normoglycemic mildly hyperinsulinemic
	obese	511.8 + 62.3*	15	21.96 + 1.97*	2.64 + 0.69*	Hyperglycemic Hyperinsulinemic

Obese ZF rats exhibited about two-fold increased fasting plasma insulin levels compared to obese Wistar rats as well as normal fasting plasma glucose levels indicative for their hyperinsulinemic insulin-resistant, but normoglycemic non-diabetic state. At variance, obese ZDF rats are characterized by moderately elevated and even lower fasting plasma insulin levels compared to obese Wistar and obese ZF rat, respectively, but suffer from fasting plasma glucose levels exceeding those of obese Wistar and obese ZF rats by about three-fold and four-fold, respectively. Thus, obese ZDF rats can be regarded as a model for pronounced hyperglycemia and

mild hyperglycemia with relative insulin deficiency, which is typical for the metabolic stage prior to further impairment and final complete loss of ß-cell function and development of frank type II diabetes. Lean ZDF rats are characterized by normal fasting plasma glucose and elevated fasting plasma insulin levels which are higher than those of the mildly hyperinsulinemic obese Wistar rats. On basis of normal but nevertheless higher fasting plasma glucose levels of the latter compared to the normoglycemic lean ZDF rats, the physiological reasons for the hyperinsulinemia may differ between the two rat groups, i.e. secondary compensation of primary insulin resistance of glucose utilization of peripheral tissues in obese Wistar rats *vs*. primary hypersecretion of insulin by defective (overactive) pancreatic ß-cells with compensation of life-threatening hypoglycemia by secondary insulin resistance of glucose uptake into peripheral tissues in lean ZDF rats. The molecular defects leading to secondary and primary hyperinsulinemia, respectively, have therefore to be allocated to muscle, adipose and liver tissues for obese Wistar and pancreatic ß-cells for lean ZDF rats.

Supplementary Figures

Supplementary Figure S1. Structure of the human GPI-AP acetylcholinesterase (AChE) and the atypical transmembrane protein (TMP) caveolin-1 (Cav1).

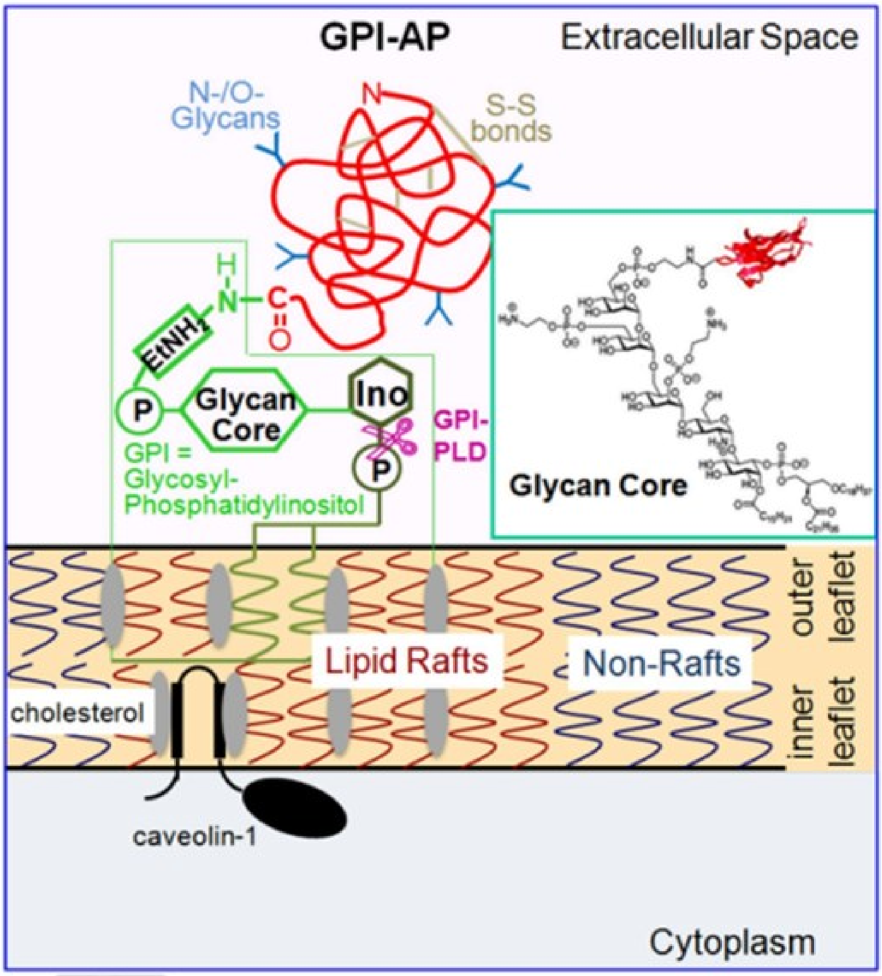

GPI-APs consist of a large polypeptide domain (red; 3D structure in the inset), typically harboring N-/O-glycosidically linked glycan chains (blue) and intrachain disulfide bonds (brown), and a GPI anchor which is constituted by a phosphatidylinositol moiety (P-Ino) with long saturated fatty acids (dark green) and a glycan core (light green; inset, black for a detailed structure for human AChE). The carboxyl terminus of the polypeptide domain and the terminal mannose residue of the glycan core are coupled *via* an ethanolamine moiety (EtNH) through amide (N-H) and phosphodiester (-P-) linkages, respectively. The fatty acids of the GPI anchor are embedded in the outer leaflet of PM, typically within lipid rafts (characterized by high cholesterol content and expression of Cav1), with the polypeptide domain protruding into the extracellular space. Cleavage by GPI-PLD (e.g. GPLD1) is indicated (pink) thereby separating the phosphatidic acid moiety left in the outer leaflet from the inositol (Ino)-glycan protein moiety released into the extracellular space.

Supplementary Figure S2. Structures of sulfonylureas (SUs) of the 1st, 2nd and 3rd generation and non-sulfonylureas. The basic sulfonylurea (tolbutamide) and benzamido (meglitinide) moieties which are fused together in a single molecule of SU of both the 2nd and the 3rd generation are indicated in yellow and green, respectively.

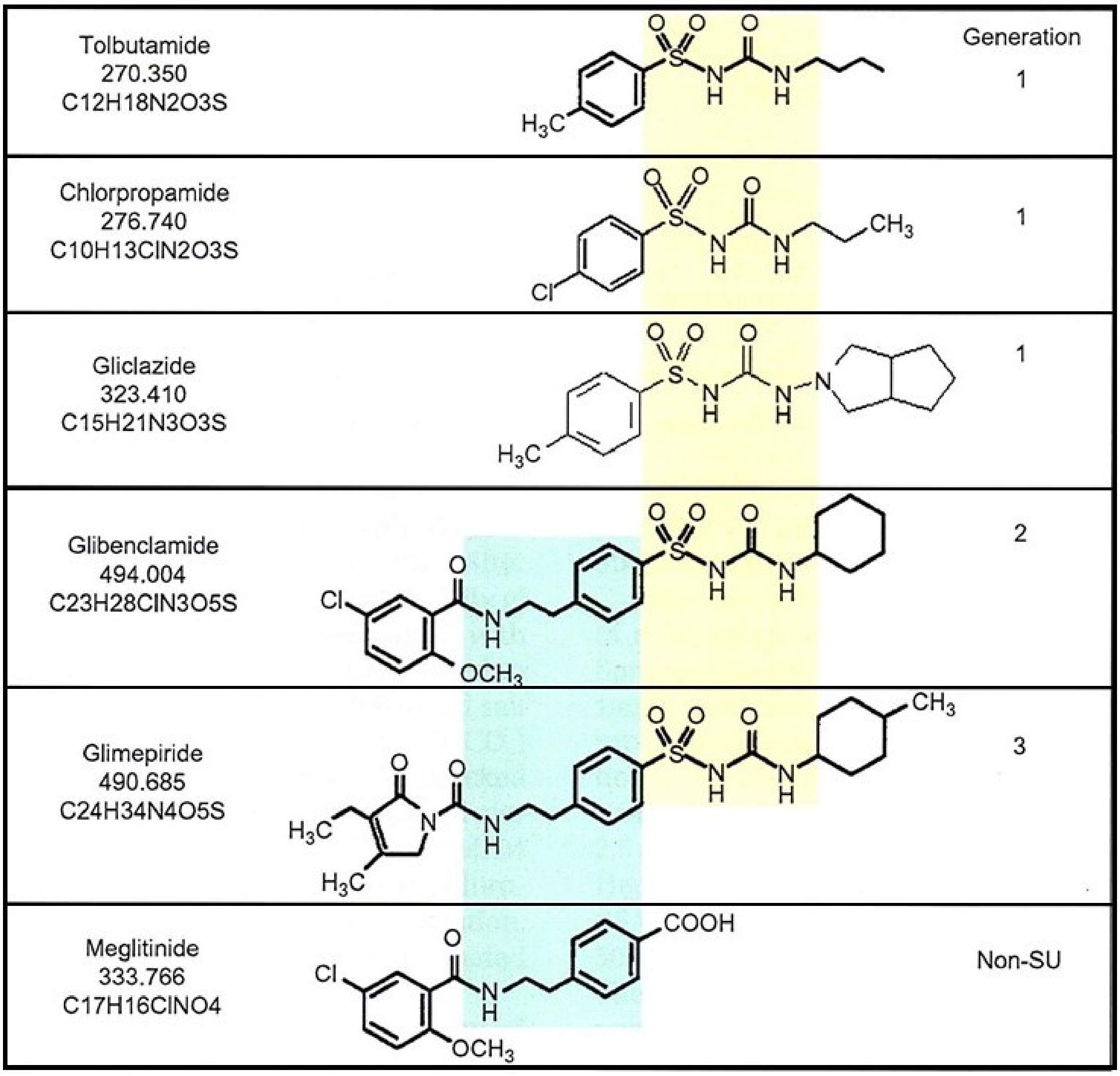

Apparently, subtle differences in the molecular structure of drugs may exert profound differences in their pharmacological profiles, as exemplified here for glibenclamide and glimepiride with the significantly higher potency for GPI-AP transfer and the resulting anabolic phenotype (stimulation of glycogen synthesis) of the latter compared to the former SU. These molecular mechanisms, the engagement of which by SUs of the 2nd and 3rd generation has been elucidated in the present study, may contribute to the more pronounced so-called extrapancreatic activity (i.e. blood glucose decrease without additional insulin release through upregulation of peripheral glucose utilization, such as glycogen synthesis) of glimepiride vs. glibenclamide, which has been demonstrated in numerous cellular, animal and clinical studies (see main text for Discussion and References). Therefore, it seemed to be justified to classify glimepiride into a novel, i.e. 3rd generation.

Supplementary Figure S3. Downregulation of the membrane-associated Cdc42, RhoA and Rac1 proteins in ELCs by silencing. Transwell co-cultures were run (37 °C, absence of serum and BSA, 2 weeks) with human adipocytes as donor and ELCs as acceptor cells in the insert and bottom wells, respectively, in the absence of siRNA (blue lines) or presence of individual siRNA directed against Cdc42 (yellow lines), Rac1 (green lines) or RhoA (turquoise lines) or a combination of all siRNAs (identical concentration each as for the individual incubations; red lines) or scrambled siRNA (black lines). Subsequently, total membranes (M) were prepared from the ELCs of the bottom wells, then coupled to the surface of chips by ionic/ covalent capture and then analyzed for expression of the membrane proteins indicated by SAW sensing as described in section 4. Phase shift Δ in response to injection of the individual anti-Cdc42, anti-RhoA, and anti-Rac1 antibodies or their combination (1100-2000 s) are indicated by horizontal hatched lines and brackets for each incubation in the absence or presence of siRNA. The experiment was repeated three to five times (different transwell co-cultures), with a representative one shown.

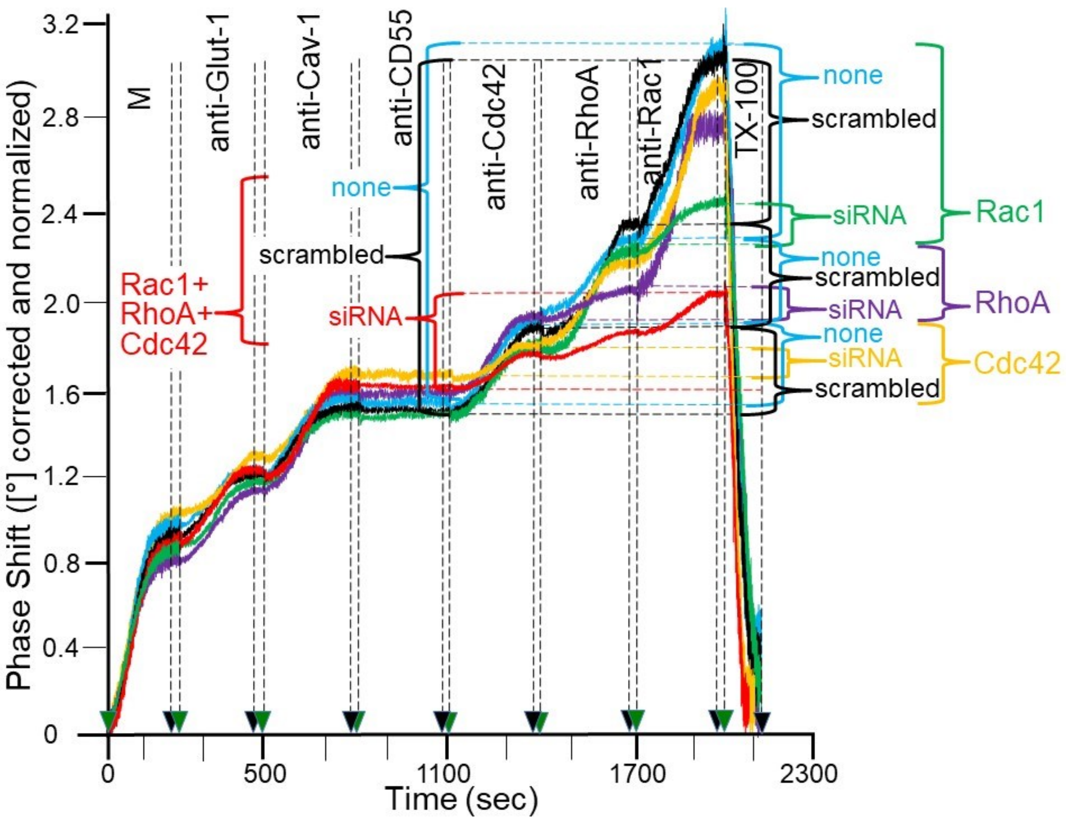

siRNAs directed against either Rac (green symbols), RhoA (turquoise symbols) or Cdc42 (yellow symbols) alone or in combination (red symbols), but not scrambled (nonsense) siRNAs (black symbols) caused downregulation of the expression of the corresponding membrane proteins in GPI-deficient EL cells to levels given in Supplementary Table S2. In contrast, expression of the typical plasma membrane protein Glut1, atypical membrane protein Cav-1 and GPI-AP CD55 was not considerably affected by silencing with the corresponding sense and scrambled (nonsense) siRNAs.

Supplementary Figure S4. Responsiveness towards insulin and SUs of human adipocytes, but not of EL cells. Human adipocytes of lipid-loading stage II [30] (**a**,**b**) or GPI-deficient human ELCs (**c**,**d**) were grown in the bottom wells of transwell co-cultures as described in section 4 and then incubated (20 min, 37 °C) in the presence of 200 µL of serum from obese ZDF rats with increasing concentrations of human insulin (**a**,**c**) or glimepiride (**b**,**d**) in the absence (red curves) or presence of 30 µM GPI2350 (yellow curves) or 5 µM wortmannin (blue curves). The adipocytes were then assayed for lipid synthesis as the incorporation of NBD-FA into total fluorescent acylglycerols during the subsequent incubation (120 min, 37 °C; quantitative evaluation of the amounts of total fluorescent acylglycerols by separation and visualization using thin layer chromatography and fluorescence imaging). The ELCs were then assayed for glycogen synthesis as described for Figure 1c,d. Lipid and glycogen synthesis in the absence of insulin (**a**,**c**) or glimepiride (**b**,**d**) is set at 1000 arb. units each. The experiments were repeated three to six times (distinct cell preparations and incubations; **a**-**d**). Significant differences between incubations in the absence and presence of insulin (**a**) or glimepiride (**b**) at each concentration (red curves) as well as absence and presence of wortmannin (blue curves) or GPI2350 (yellow curves) at each insulin (**a**) and glimepiride (**b**) concentration are indicated by blue and yellow symbols, respectively (means ± S.D.; * $p \leq 0.01$, $^{\#}$ $p \leq 0.02$, § $p \leq 0.05$).

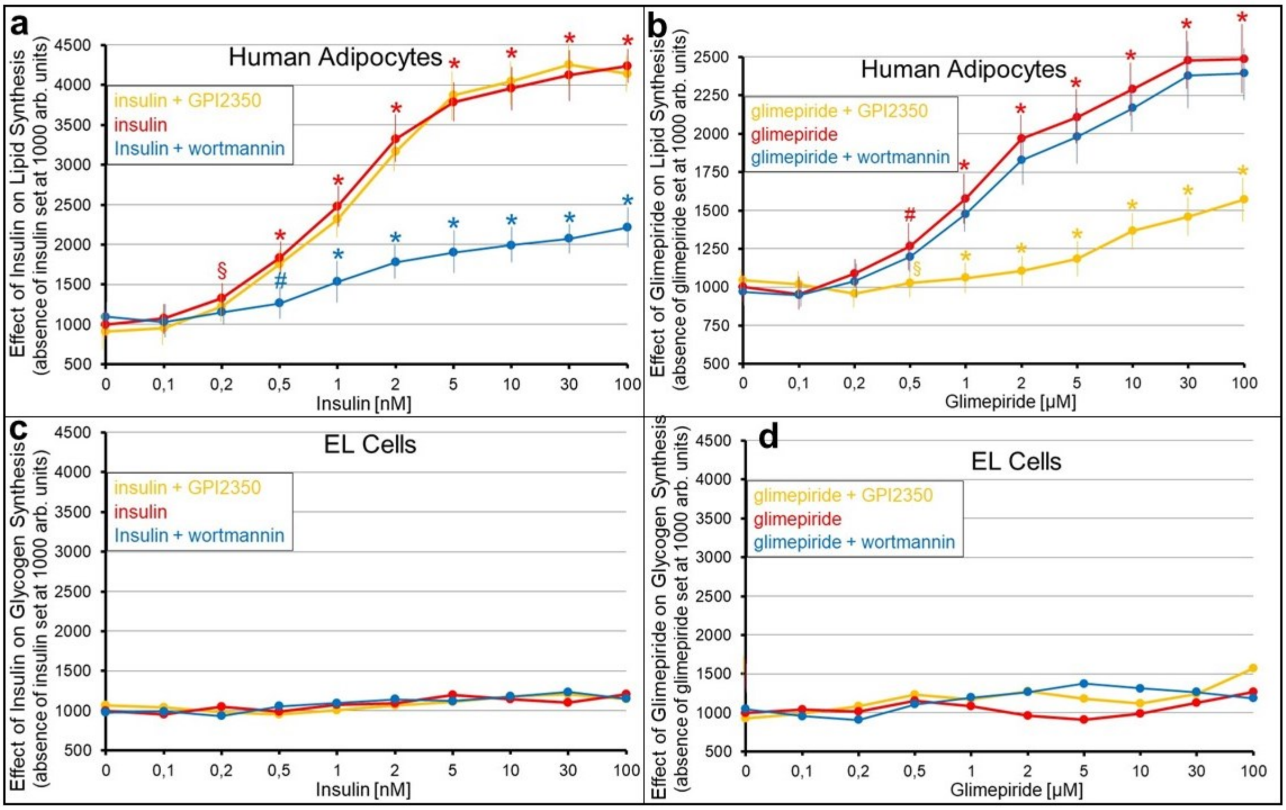

Human insulin and glimepiride managed to activate lipid synthesis in cultured human adipocytes in concentration-dependent fashion to up to 4.3-fold and 2.5-fold, respectively, with EC_{50} of 0.9 nM

(Supplementary Figure S4a) and 1.6 µM (Supplementary Figure S4b). Blockade of insulin, but not glimepiride action by wortmannin, a specific non-competitive inhibitor of phosphatidylinositol-3' kinase inhibitor, which represents a central component of canonical insulin signaling (see section 3), and vice versa abrogation of glimepiride, but not insulin action by the GPI-PLC inhibitor GPI2350, a critical component of the insulin-mimetic signaling of glimepiride in peripheral insulin target cells, strongly argue for the engagement of different signaling mechanisms to the lipid synthesizing machinery by insulin and glimepiride (see section 3), which are initiated by the occupied insulin receptor and lipid rafts with intercalated glimepiride, respectively. In contrast, both insulin and glimepiride failed to upregulate glycogen synthesis in GPI-deficient (Supplementary Figure S4c,d) as well as wildtype (Müller and Müller, unpublished results) ELCs to any measurable extent. This presumably relies on the missing expression of critical components of the insulin as well as glimepiride signaling pathways in the ELCs, possibly the insulin receptor and the GPI-PLC, respectively. In fact, it has previously been demonstrated that gain of insulin responsiveness of glycogen synthesis in wildtype ELCs critically depends on their prior transfection with insulin receptor cDNA [35,63].

Importantly, glimepiride, but not insulin, stimulation of lipid synthesis in human adipocytes was strictly dependent on the presence of serum (with that from obese ZDF rats being most efficiently). Lack of serum caused complete failure of glimepiride, but not insulin, to upregulate lipid synthesis (Müller and Müller, unpublished data). This finding is explained best by operation of the "indirect" mode of GPI-AP transfer with accompanying anabolic effect, i.e. increase in lipid synthesis, as the underlying molecular mechanism for the induction of the so-called extrapancreatic activities (i.e. stimulation of glucose utilization without additional insulin release) of SUs of the 2nd and 3rd generation. Apparently, full-length GPI-APs loaded onto serum GPI-binding proteins are transferred to acceptor adipocytes upon their displacement by PIG-proteins, which have been generated by SU-dependent GPI-PLC by cleavage of full-length GPI-APs at the PMs of donor adipocytes. Thus, this "extracellular" signaling of glimepiride, initiated by redistribution and activation of lipid raft components, among them insulin-/SU-dependent GPI-PLC, of SU molecules intercalated into PM rafts, rather than a tyrosine phosphorylation-dependent pathway induced by a raft-associated and glimepiride-activated intracellular non-receptor tyrosine kinase to canonical insulin signaling (i.e. insulin receptor substrate proteins), as has been suggested some years ago, seems to be involved in the anabolic regulation of glucose and lipid metabolism by SUs of the 2nd and 3rd generation, in particular by glimepiride.

Supplementary Figure S5. Structures of PIG1, 7, 37 and 41. PIG41 corresponds to HO-SO_2-O-6Manα1(Manα1-2)-2Manα1(6-HSO_3)-6Manα1-4GluNβ1-6(D)-inositol-1,2-(cyclic)phosphate, PIG37 to HO-PO(H)O-6Manα1(Manα1-2)-2Manα1-6Manα1-4GluNβ1-6(D)-inositol-1,2-(cyclic)-phosphate, PIG45 (structure not shown) to Manα1-2Manα1-2Manα1(6-HSO_3)-6Manα1-3GluNβ1-3(D/L)inositol-6-sulfate, PIG7 to Manα1-4GluNα1-6(L)inositol-1,2-(cyclic)-phosphate and PIG1 to HO-PO(H)O-6Manα1-4GluNα1-6(L)inositol. Incubation of primary rat adipocytes with PIGs resulted in stimulation of glucose transport via Glut4 translocation to PMs and lipid synthesis in concentration-dependent fashion with PIG41 being most potent, followed by PIG37, PIG45, PIG7 and PIG1, in that order of declining efficacy. Chemically synthesized PIGs have intensely been tried to use as insulin-mimetic agents for the treatment of type II diabetes. However, despite their considerable insulin-mimetic activities reaching to up to 60% of the maximal insulin effect and huge efforts of pharmaceutical industry in varying their structure, PIGs finally failed to achieve this goal due to their large size, which prevented oral bioavailability and their carbohydrate nature with glycosidic linkages, which interfered with their stabilization against hydrolytic degradation in the stomach and intestine. Reduction of size and replacement of sugar moieties and glycosidic bonds using modern techniques of rational drug design and chemical biology turned out to be not successful.

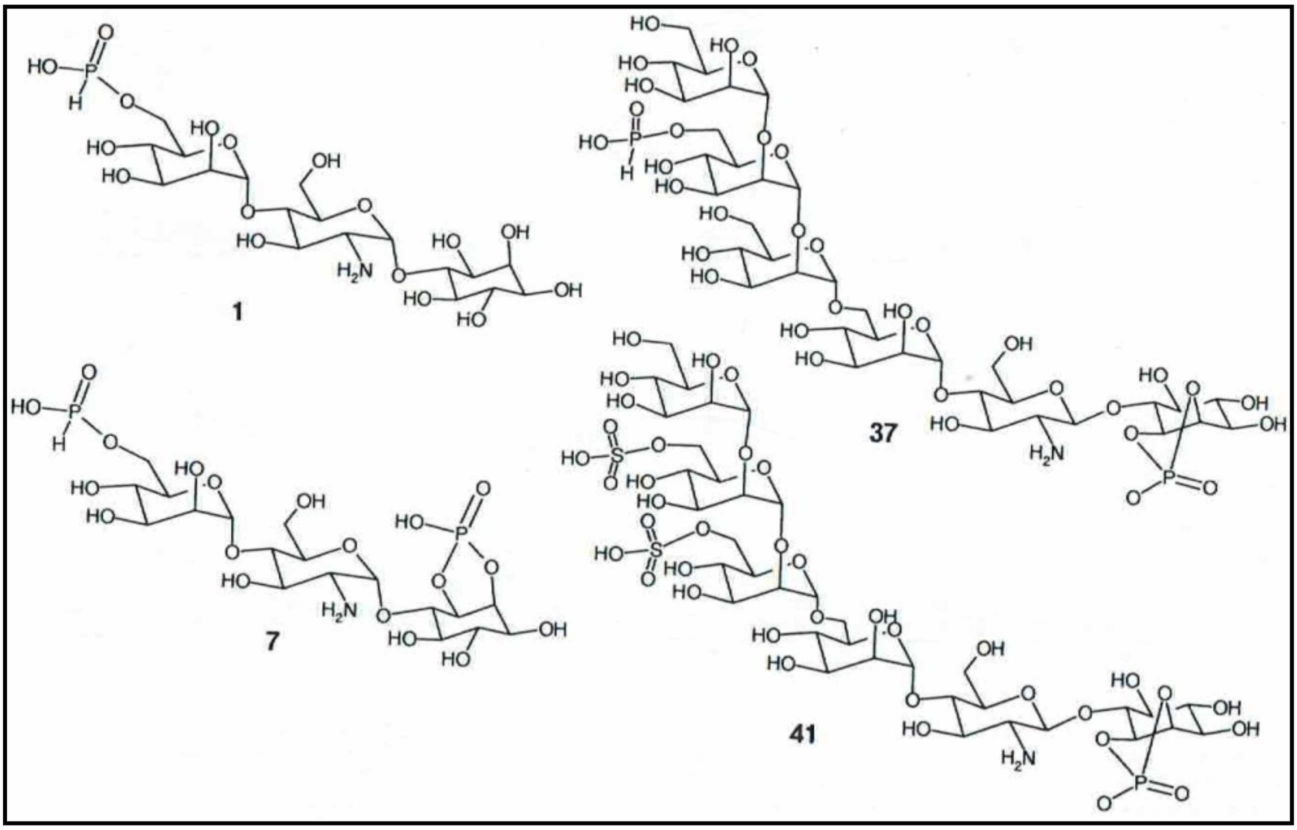

As a byproduct of this study, its data may help to understand the heavily debated controversy about the operation of PIGs as second messengers of insulin action, which started in 1986 and

lasted for more than two decades, at least in part. In fact, a role in insulin-mimetic signaling has been attributed to naturally occurring PIGs as the lipolytic (and proteolytic) degradation products of specific GPI lipids (and GPI-APs). This was based mainly on their potential to stimulate glucose and lipid metabolism in insulin target cells, such as myocytes and adipocytes, upon incubation of PIG(-peptides) of defined structure in vitro. However, subsequent problems with the reproducibility of some of these results as well as the size of the effects yielded with different PIG preparations (including those of unknown structure) and different assay systems by various laboratories discredited this hypothesis.

The findings presented in previous studies suggested that considerable variation in the efficacy of structurally defined PIGs is explained by differential responsiveness of the target (in particular adipose and muscle) cells towards PIGs and insulin. With regard to adipocytes, specific requirements for cell size and degree of lipid-loading as well as specific conditions for their preparation (i.e. collagenase digestion) and incubation (i.e. shaking intensity, titer, presence of albumin or serum) have to be fulfilled, which apparently are not identical with those supporting maximal insulin action. For instance, defatted BSA (primary cells) or certain serum fractions (cultured cells), which are typically used for cell biological experiments, support insulin activity, however interfere with the detection of PIG insulin-mimetic activity, i.e. the higher their concentration the higher the insulin and the lower the PIG activity. It is likely that detection of the latter strictly depends on serum albumin or specific serum proteins loaded with full-length GPI-APs, which are removed in course of routine procedures for commercialization, such as defatting. Exact description of the assay components as well as tight control of the experimental conditions are therefore of tremendous importance to guarantee valid results and correct conclusions about PIGs as functionally relevant molecules in physiologically relevant studies in the future (for a more detailed discussion of this topic and references see [30]).
